# Recent Progress in Rapid Determination of Mycotoxins Based on Emerging Biorecognition Molecules: A Review

**DOI:** 10.3390/toxins14020073

**Published:** 2022-01-20

**Authors:** Yanru Wang, Cui Zhang, Jianlong Wang, Dietmar Knopp

**Affiliations:** 1 College of Food Science and Engineering, Northwest A&F University, Yangling, Xianyang 712100, China; yanruwang@nwafu.edu.cn (Y.W.); cuizhang@nwafu.edu.cn (C.Z.); 2 Chair for Analytical Chemistry and Water Chemistry, Institute of Hydrochemistry, Technische Universitat München, Elisabeth-Winterhalter-Weg 6, D-81377 München, Germany

**Keywords:** mycotoxins, antibodies, aptamers, short peptides, molecularly imprinted polymers, rapid tests, lateral flow assay, microplate assay, biosensor, multiplexing

## Abstract

Mycotoxins are secondary metabolites produced by fungal species, which pose significant risk to humans and livestock. The mycotoxins which are produced from *Aspergillus*, *Penicillium,* and *Fusarium* are considered most important and therefore regulated in food- and feedstuffs. Analyses are predominantly performed by official laboratory methods in centralized labs by expert technicians. There is an urgent demand for new low-cost, easy-to-use, and portable analytical devices for rapid on-site determination. Most significant advances were realized in the field bioanalytical techniques based on molecular recognition. This review aims to discuss recent progress in the generation of native biomolecules and new bioinspired materials towards mycotoxins for the development of reliable bioreceptor-based analytical methods. After brief presentation of basic knowledge regarding characteristics of most important mycotoxins, the generation, benefits, and limitations of present and emerging biorecognition molecules, such as polyclonal (pAb), monoclonal (mAb), recombinant antibodies (rAb), aptamers, short peptides, and molecularly imprinted polymers (MIPs), are discussed. Hereinafter, the use of binders in different areas of application, including sample preparation, microplate- and tube-based assays, lateral flow devices, and biosensors, is highlighted. Special focus, on a global scale, is placed on commercial availability of single receptor molecules, test-kits, and biosensor platforms using multiplexed bead-based suspension assays and planar biochip arrays. Future outlook is given with special emphasis on new challenges, such as increasing use of rAb based on synthetic and naïve antibody libraries to renounce animal immunization, multiple-analyte test-kits and high-throughput multiplexing, and determination of masked mycotoxins, including stereoisomeric degradation products.

## 1. Introduction

Mycotoxins are secondary metabolites produced by different species of filamentous fungi, including *Aspergillus*, *Fusarium*, *Penicillium*, *Alternaria*, *Claviceps*, etc. [1,2,3]. They can be found in various food and feed, such as cereals, nuts, oilseeds, fruits, spices, coffee, wine, beer, and foods of animal origin, including dairy products, meat, and eggs [4,5,6,7,8]. Mycotoxin contamination in both food and feed commodities is considered to be inevitable due to the widespread occurrence of mycotoxin-producing fungi in the environment [9]. Although more than 400 mycotoxins with diverse structures have been identified, a limited number of compounds are considered a problem in food and feed safety. These include aflatoxins (AFs) [10,11], ochratoxin A (OTA) [12,13], fumonisins (FMs) [14], T-2/HT-2 toxins [15,16], deoxynivalenol (DON) [17,18], zearalenone (ZEN) [19,20], citrinin (CIT) [21], patulin (PAT) [22,23], and ergot alkaloids (EAs) [24,25] due to their significant prevalence in food and feed and severe health risks to humans and animals.

Among these mycotoxins, AFs have received the most attention due to their high toxicity. Aflatoxin B1 (AFB1) has been classified as Group 1 agent (potent human carcinogen) by the International Agency for Research on Cancer (IARC) of World Health Organization (WHO). It has long been associated with liver cancer, and more recent researches have exposed its negative role in nutrition outcomes and immune suppression effects [26]. OTA (IARC 1993) and fumonisin B1 (FB1) (IARC 2002) are suspect human carcinogens, which are classified as Group 2B agents (possibly carcinogenic in humans) [27]. The presence of other mycotoxins in diet has also been demonstrated to cause adverse and chronic health effects, such as gastrointestinal symptoms (DON) [28], endocrine-disrupting effects (ZEN) [29], growth retardation (DON, T-2 toxin) [30,31], nephrotoxicity (CIT) [32], and genotoxicity (DON, CIT, PAT) [33,34,35]. Furthermore, co-exposure of several mycotoxins to humans and animals through diet may cause additive or synergistic effects, which have been reported in studies using cell cultures and animals [36,37,38]. Table 1 lists the major mycotoxins and their main producing fungi species, affected food commodities, and toxic effects to humans and animals.

Owing to their poisonous character and widespread prevalence in food and feed products, maximum permitted levels (maximum residue limits, MRLs) for most toxic mycotoxins in multiple food and feed products have been set worldwide. The limit values differ among countries as well as to related commodities. Table 2 compares the maximum permitted levels of major mycotoxins in food as set by the European Union (EU), the United States (U.S.), and China. Among all the food commodities, infant foods have the lowest permitted levels for all mycotoxins. 

To address the legislation and ensure food safety, the development of analytical methods with high sensitivity and accuracy is of great demand. Current analytical methods include confirmatory methods and screening methods. The standardized methods for mycotoxin analysis are chromatographic methods, including thin-layer chromatography (TLC), gas-chromatography (GC) with electron capture detection (ECD) [48], flame ionization detection (FID) [49] or mass spectrometry (MS) [50], high performance liquid chromatography (HPLC) with ultraviolet detection (UV) [51], fluorescence detection (FLD) [52], and MS or tandem mass spectrometry (MS/MS) [53,54]. TLC was the predominant method in early days. Although it is still used by some laboratories, it has almost been replaced by HPLC and GC. The instrumental methods are usually used as the gold standard. Nevertheless, despite their accurate and precise determination, sophisticated instrumental methods have some limitations related to high cost, long detection time, and the requirement of skilled operator [55,56]. 

In response to these limitations, a couple of rapid methods with high sensitivity and specificity have been developed for the identification and quantification of mycotoxins [57,58,59,60,61]. Furthermore, researchers are still working on developing novel methods with improved sensitivity, specificity, robustness, time-saving, and cost-efficiency. Rapid methods are more preferred by analysts who need to know the results immediately (e.g., on-site screening of high numbers of samples) or in routine analysis in laboratories where the classical method is not available. Among all the rapid detection methods for mycotoxins, immunoassays have already found widespread use as screening methods, providing beneficial attributes, such as rapidness, simplicity, cost-efficiency, required sensitivity, and specificity [62,63,64,65].

**Figure 1:**
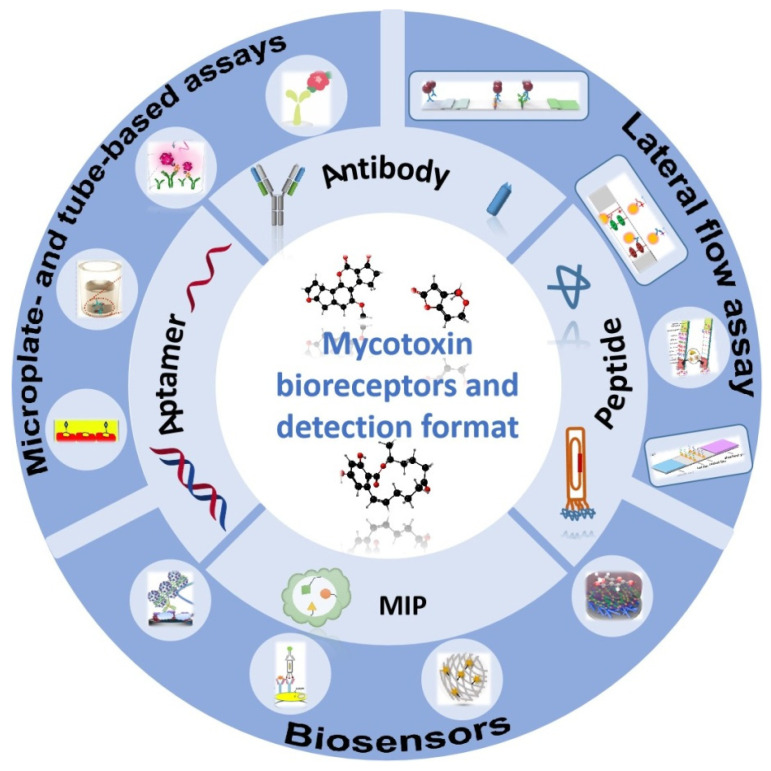
Schematic illustration of mycotoxins recognition elements and their application.

The core principle of immunoassays is the molecular interaction between target and biorecognition element, i.e., the antibody. So far, antibodies have been regarded with no doubt as the gold-standard recognition element in immunoassays and biosensors. Polyclonal and monoclonal antibodies dominate the field. However, the development of molecular techniques for expression of complete antibodies or antibody fragments in different species and methods for production and screening of combinatorial libraries is challenging. It has opened a wide range of opportunities for the selection of rAbs and their engineering, i.e., production of tailored binders with predefined properties in different species, e.g., bacteria, yeast, and mammalian cells (Chinese hamster ovary, CHO cells). In addition, plants and crop species offer the necessary economy and scalability to enable extremely cost-effective and efficient production of antibodies (plant-based antibodies) [66,67,68]. Besides rAbs, other novel recognition elements are emerging in recent decades, including aptamers [69], short peptides [70], and molecularly imprinted polymers (MIPs) [71,72,73]. These reagents have the potential to overcome some of the disadvantages of conventional antibodies, e.g., stability and production issues. Considering the increasing number of emerging rapid methods for mycotoxin detection, it is important critically discuss the differences between the used biorecognition molecules and arising advantages and disadvantages of their application. Thus, in this review, we provide an overview of the current and emerging biorecognition molecules towards mycotoxins and discuss their strengths and weaknesses for mycotoxin monitoring (Figure 1). Furthermore, we also introduce the application of those recognition elements in various assay formats, e.g., microplate- and tube-based assays, lateral flow assays (LFA), immunoaffinity columns (IAC), and biosensors. 

## 2. Biorecognition Molecules

### 2.1. Antibodies 

Among all the biorecognition molecules, antibodies are the most popular and widely applied due to their superiority in terms of affinity and specificity. There are mainly three types of antibodies, including pAb, mAb, and rAb. Affine polyclonal antibodies can be prepared in a relatively short period (around 10–12 weeks) at low cost. The first pAbs for mycotoxin detection were reported nearly 40 years ago [74,75]. In these publications, polyclonal antibodies were produced by simply collecting the serum of a New Zealand rabbit after several injections of antigens. PAbs are a mixture of antibodies towards different determinants of the antigen. Thus, they have disadvantages related to inconsistence among different antibodies of the same batch and between batches. Further, it is impossible to prepare pAbs with same characteristics using the identical reagents and immunization schedule but a different animal. This is almost a deal-breaker for long-term and higher sales commercial exploitation. However, pAbs have been and are still being widely applied in mycotoxin determination due to the benefits of ease of development, short production period, and relatively low cost [76,77,78,79]. 

In 1975, Köhler and Milstein invented the hybridoma cell technology, which allows the production of homogenous antibodies [80]. By hybridizing antibody-producing B-lymphocytes with myeloma cells, a hybridoma cell line can be selected and isolated. MAbs then can be produced by cultivation of hybridoma cells either in vivo or in vitro. Since the first mAbs described for AFs [81], AFM1 [82], OTA [83], DON [84], ZEN [85], T-2 toxin [86], and FMs [87], numerous mAbs have been developed and applied in both laboratory research and commercial assay products [88,89,90,91,92]. 

**Figure 2:**
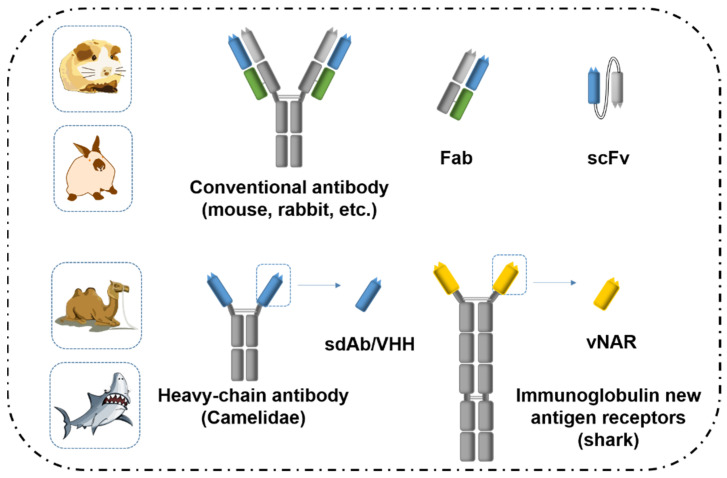
Schematic diagram of antibody structure. Abbreviations: Fab, antigen binding fragment; scFv, single-chain variable fragment; sdAb, single-domain antibody; VHH, variable domain of heavy chain of HCAb; vNAR, variable domain of new antigen receptors.

With the advancement of genetic engineering, the third generation of antibodies, named rAb technology, emerged [93]. Conventional IgG antibodies (MW 150 kDa) are composed of two identical heavy chains (50 kDa) and two identical light chains (25 kDa), which are linked together by disulfide bonds (Figure 2). It is a Y-shaped, multidomain protein with antigen-binding sites located on the complementarity determining regions (CDRs) of the variable domains of the heavy and light chains. Cloning and expression of the antibody variable domains in prokaryotic or eukaryotic systems can produce rAbs reproducibly and steadily. A wide variety of rAbs have been produced, including antigen binding fragment (Fab) [94], single-chain variable fragment (scFv) [95,96,97], and single-domain antibody (sdAb) [98,99]. For rAb development, antibody binding genes either from lymphocytes of the immunized animal or from hybridoma cells are cloned and displayed on phages [100,101], bacteria [102,103], yeast [104], or mammalian cells [105,106,107]. Acellular approaches use ribosome or mRNA display. Phage display is the most used technology for in-vitro rAb development. Antigen binding fragments can be enriched after 4–5 rounds of biopanning. The most powerful advantage of biopanning technique is that an antibody can be obtained with desired selectivity or affinity through optimization of panning conditions. Compared with conventional antibodies, rAbs can be produced at a lower cost, with higher consistence and smaller size, and without the use of animals. Single-chain antibodies towards mycotoxins have been successfully expressed in bacteria and yeast [108,109,110,111,112,113]. However, Fab and scFv antibody fragments are usually suffer from instability and low production yield, which are the major limiting factors of this technology. 

In the serum of Camelidae and cartilaginous fish is a considerable fraction of heavy-chain antibodies (HCAbs), which lack the light chains (Figure 2) [114,115]. While HCAbs of Camelidae lack the CH1 domain, that of cartilaginous fish, also called immunoglobulin new antigen receptors (IgNARs), have five constant domains. Thus, the variable domain of the heavy chain is linked directly to the hinge region in HCAbs. The antigen-binding fragment (~15 kDa) of the HCAbs, which constitutes only the variable domain of the heavy chain (VHH from camels and llamas; VNAR from sharks), is a single-domain antibody (sdAb), also called nanobody [116]. Compared with conventional antibodies and antibody fragments, including scFv and Fab, nanobodies have higher thermostability and solvent-resistance. The interloop disulfide bond in camelid VHH was considered to contribute strongly to its high stability and thermostability [117,118]. The presence of several amino acid substitutions in the framework region 2 provide VHH a more hydrophilic and soluble character. In most publications, the thermostability of nanobody was verified by testing its binding ability after treatment at extreme temperatures for various periods and comparing with pAb/mAb. The anti-idiotypic nanobody towards OTA developed by Zhang et al. [119] has enhanced thermostability compared to a mAb. The VHH retained more than 50% of its activity after being heated at 80 °C for 40 min, whereas the mAb lost most of its binding ability after 10 min incubation at the same temperature. Liu et al. [120] developed four different nanobodies against OTA. All nanobodies showed higher thermostability than mAb 6H8. Among them, Nb 32 is the most stable one, which could stand at 95 °C for 5 min without loss of its activity and retained 50% of its binding ability after incubation at 90 °C for 75 min. He et al. [121] developed a nanobody towards AFB1 and evaluated the solvent tolerance towards MeOH, DMSO, DMF, acetone, and acetonitrile. The data indicated the VHHs demonstrated higher resistance to MeOH than mAbs. Separate from mycotoxins, in a study with the herbicide parathion reported by Zhang et al., VHH9 could maintain nearly half of its binding activity under 40% of MeOH, DMSO, and acetonitrile [122]. Above all, nanobodies are superior biorecognition reagents compared with conventional antibodies, scFv and Fab fragments, which are less prone to loss of activity at high temperatures or in complex sample composition. 

However, there are also some drawbacks in the development of nanobodies. First, camelid animals are not as easy to grow as small animals, such as mice, rabbits, or chicken. For that matter, using transgenic mice for immunization or panning of naïve or synthetic nanobody libraries might be an outcome [123]. Second, it is not easy to obtain a nanobody with high affinity, especially for small molecules. Up to now, mycotoxin-specific nanobodies were developed only towards AFs [121], OTA [120], 15-acetyl-deoxynivalenol [124], and tenuazonic acid [125]. The limited availability constitutes a clear shortage for the development of multi-mycotoxin assays. Third, due to its small size, the nanobody’s random attachment to surfaces (e.g., polystyrene plate, nitrocellulose membrane, nanomaterials) can negatively impair its binding affinity [126,127]. The binding sites of the nanobody are more likely to be hindered sterically after immobilization compared with that of IgG. 

Affinity and specificity are two important parameters for antigen binding probes. Preparation and designation of effective mycotoxin antigens that contain characteristic structure and could be exposed to the body is essential for successful isolation of specific and highly affine functional antibodies. Mycotoxins are small molecules (MW < 1000), which must be conjugated with a carrier protein in order to elicit an immune reaction. The structure of commonly used mycotoxin antigens and obtained antibody characteristics are summarized in Table 3. Mycotoxins have different functional groups, and therefore, a variety of coupling strategies were utilized. OTA, FB1, and CIT all have a carboxyl or amino group that can be activated and coupled to amino groups of carrier proteins to form stable amide linkages. AFs do not have an activatable group for direct conjugation with protein. Most established is the conjugation of AFB1 to a protein, such as keyhole limpet hemocyanin (KLH), bovine serum albumin (BSA), or ovalbumin (OVA), in the 1-position by means of a carboxymethyloxime (CMO) spacer. By far, most of all aflatoxin selective antibodies produced over the last decades have been generated by immunizations with this (commercially available) conjugate. The resulting antibodies all show similar selectivity. The affinity to the four major AFs usually follows the order AFB1 > AFG11 > AFB2 > AFG2 [128,129,130,131,132]. The immunization schedule and antibody screening techniques also have an important effect on the quality of resultant antibodies. By using a rapid cell fusion technique, Wu et al. [133] selected a cell line from 100,000 positive cell clones, which produced an mAb with similar recognition ability for AFB1, AFB2, AFG1, AFG2, AFM1, and AFM2. Devi et al. generated 10 hybridomas by immunizing AFB1-oxime-BSA to mice with an alternative immunization protocol [134]. One of them was highly specific to AFB1 because it only showed a weak cross-reaction with AFG1 (12%). High-affinity broad spectrum [135,136] and AFB1-specific [137,138,139] aflatoxin antibodies could also be generated using AFB2-conjugates that are less toxic than AFB1-conjugates. Synthesis of DON-protein conjugate was carried out mostly by converting the C3 hydroxyl group to carboxyl [84,140,141,142]. Similarly, T-2 antigen was prepared by esterification of the C3 hydroxyl group to obtain T-2-hemisuccinate (3-HS-T-2) [143,144,145,146,147]. 

Most of anti-ZEN antibodies reported were obtained by immunization with zearalenone-6′-carboxymethyloxime-protein conjugate [89,148,149,150,151,152]. Due to the protein binding position, those developed antibodies could not discriminate carbonyl and hydroxyl functional groups at position C6′ and thus usually showed high cross-reactivity with ZEN derivatives (including α-zearalenol, β-zearalenol, zearalanone, α-zearalanone, and β-zearalenone). To produce a specific antibody towards ZEN, Teshima et al. synthesized 5-aminozearalenone by a two-step approach and coupled it with protein at C-5 position of the compound [153]. The anti-ZEN mAb exhibited high specificity to ZEN, with weak cross-reactivity (<4%) to other analogs. Gao et al. coupled ZEN with cationic bovine serum albumin (cBSA) via a Mannich reaction [154]. By using this immunogen, specific anti-ZEN pAbs and mAbs were obtained, with cross-reactivity less than 7%. Sun et al. generated mAbs towards ZEN with a novel ZEN-BSA conjugate, which was prepared using 1,4-butanediol diglycidyl ether as a linker [155]. The selected antibody showed 53% cross-reactivity with zearalanone but weak cross-reactivity (<4%) with the other four analogs. 

ZEN exists in two stereoisomeric forms: *trans*- and *cis*-zearalenone. *Trans*-ZEN is known naturally produced by *Fusarium* spp. and could isomerize to *cis*-ZEN photochemically, i.e., by UV light irradiation. The coexistence of *trans/cis*- ZEN has already been reported in edible oil [156], grains, and their products [157]. However, owing to the limited study of *cis*-ZEN, worldwide maximum levels for ZEN in food and feed are thus based on the *trans*-isomer. However, toxicological studies revealed an elevated estrogenic activity of *cis*-ZEN and/or its reductive metabolites α/β-*cis*-zearalenol compared to their respective *trans*-isomers [158]. To the best of our knowledge, there were neither studies performed to discriminate between both isomers based on bioanalytical methods nor reported stereoselective antibodies towards ZEN. At an earlier stage, we reported on first experimental evidence for an enzyme-generated chemiluminescence-induced *trans-cis* isomerization of chip-immobilized *trans*-ZEN in a microfluidic cell of a biosensor using a ZEA-mAb [159]. After that, the cross-reactivity of five commercially available anti-ZEN mAbs was tested with both isomers by competitive ELISA on microplates. Dependent on the source of the antibody, significantly reduced affinity of *cis*-ZEN was obtained (CR 12–72%) (data not published). This could be well explained by the fact that only *trans*-ZEN is commercially available for synthesis of the immunogen and generation of ZEN-antibodies. As consequence, the practical use of antibody-based assays, such as immunoassays, lateral flow assays, and immunoaffinity cartridges, may result in an underestimation of real ZEN content in samples that contain appreciable amount of *cis*-ZEN. Thus, suppliers of related assays are urgently requested to include the CR of *cis*-ZEN in the assay instructions for more reliable results interpretation. Moreover, the development of antibodies for stereospecific targeting of chiral haptens like ZEN is a special research challenge.

Unlike other toxins, PAT is highly unstable and can decompose during the course of protein conjugation. Furthermore, free PAT or bond-exposed epitope of immunogen are highly reactive with nucleophiles, which could bind with thiol and amino groups of proteins covalently and thus interfere with the generation of affine antibodies. For these reasons, high specific and affine anti-PAT antibodies are rarely reported. There are mainly two approaches to synthesize the immunogen. One is based on modification of the hydroxyl function [165,167]. By reaction with glutaric anhydride, PAT was converted to PAT hemiglutarate (PAT-HG) and then coupled with a carrier protein. However, no or slight competitive displacement by free PAT was observed with pAbs using PAT-HG as immunogen. The other approach is to synthesize a PAT derivative (PAT-SAT) from L-arabinose, which lacks the highly reactive C3-C4 double bond while maintaining the original skeleton of the toxin, and then conjugate it to protein [166,168]. PAbs that were produced using this antigen showed a high titer and inhibition effect by addition of free toxin. The competitive assay could detect PAT as low as 0.06 μg/L.

Even when using the same immunogen for antibody generation, in some cases, the obtained affinity of pAbs and mAbs is not identical. Taking OTA as an example, Reddy et al. developed pAbs by injecting OTA-BSA conjugate into a New Zealand rabbit [77]. The 50% inhibition binding (IC_50_) of OTA was 5 ng/mL determined by indirect competitive ELISA (icELISA). Anti-OTA mAbs developed with the same antigen have higher affinity with IC_50_ around 0.3 ng/mL [169,170]. The single-domain antibody towards OTA developed by Liu et al. after immunization of an alpaca also had a good performance, with IC_50_ of 0.74 ng/mL and K_D_ value of 0.039 nM [120]. In most cases, scFv fragments derived from hybridoma cells have lower affinity than the parental mAb [171,172]. The scFv against AFB1 prepared by Min et al. retained 17 times less and anti-FMB1 scFv about 12-fold lower binding affinity than the parental mAbs [173]. However, one of the most powerful advantages of rAb development is that the affinity and selectivity of antibodies can be improved through in-vitro biopanning. Hu et al. [109], by using stringent panning conditions, isolated a scFv towards FMs with an 82-fold higher binding affinity than its parent mAb. There are also other factors that affect the quality of rAbs, such as the immune response of individual animals, immunization protocol, cell fusion technique, etc. Another benefit of rAb is the ease of gene modification, which could facilitate the directed evolution of antibodies [174]. Based on an anti-OTA nanobody, X. Wang et al. constructed a mutation library after identification of key amino acids of the antibody binding sites by homology modeling, molecular docking, and alanine scanning [175]. A mutant nanobody was then obtained by biopanning, which exhibited a K_D_ value of 52 nM, which is 1.4-fold and 1.36-fold lower than that of the original nanobody, respectively.

### 2.2. Aptamers

**Figure 3:**
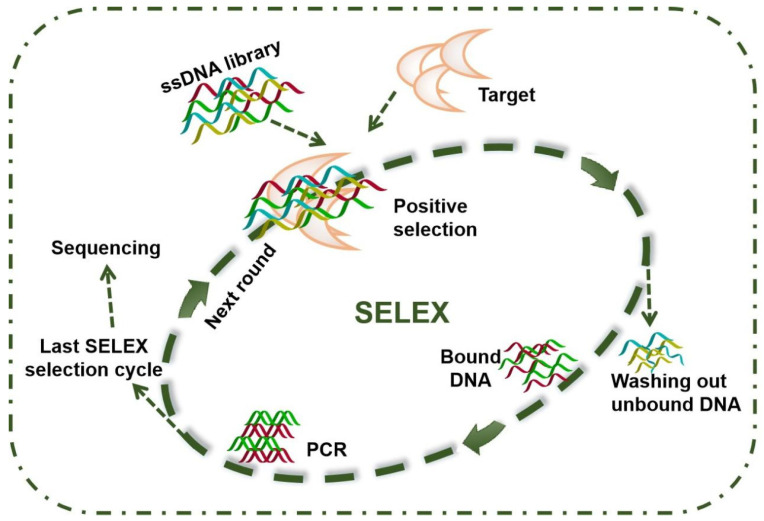
Selection of specific aptamers by SELEX technology.

Aptamer ligands are short, single-stranded DNA or RNA sequences that adopt specific three-dimensional conformations and thus can bind the target specifically in a similar way to antibodies. Since they have been raised first in early 1990s [176,177], aptamers have attracted increasing attentions due to their advantages over antibodies in terms of robustness and cost-effectiveness. Antibodies are generally obtained from biological samples, while aptamers can be synthesized in vitro in a large quantity and at low cost once the sequence is determined [178]. Furthermore, aptamers exhibit higher stability under most environmental conditions and can resist chemical and physical denaturation without losing their binding activities. Aptamers are obtained by in-vitro screening of oligonucleotide libraries through systematic evolution of ligands by the exponential enrichment process, named SELEX. As shown in Figure 3, the SELEX technique starts with a large random oligonucleotide library (with 10^14^ to 10^15^ random sequences), and each oligonucleotide contains a random central region of 20 to 80 nucleotides, flanked by two fixed primer-binding regions on 3′ and 5′ ends. During selection, the targets are immobilized on a solid surface and incubated with the library. Then, free oligonucleotides are separated, and bound ones are eluted for enrichment by PCR and used for the next round of SELEX. After 7 to 30 rounds of SELEX, the enriched pool is cloned, sequenced, and characterized to select aptamers with desired properties. Aptamers recognize the targets by a combination of van der Waals forces, hydrogen bonding, electrostatic interaction, stacking interactions and shape complementarity, which is similar to antibody-antigen recognition [178].

Aptamers with high affinity are not always easy to obtain. The diversity of the initial oligonucleotide library is crucial to obtain high-affinity aptamers. More oligonucleotide sequences lead to higher chances to obtain useful target-specific aptamers. However, in fact, the starting library diversity is always limited due to synthesis technology and nucleotide preference. Besides, some sequences may get lost during PCR amplification, and the resultant sequences do not always have the desired binding affinities towards the target [179]. Another factor that impacts the aptamer’s affinity is selection condition, including the amount of target, incubation condition, and the way to separate unbound oligonucleotides. Increasing selection pressure during SELEX can remove molecules with low affinity.

Over the past decade, aptamers towards a variety of mycotoxins have been developed. Cruz-Aguado and Penner prepared an aptamer towards OTA, which was the first aptamer identified for the detection of a mycotoxin [180]. The selected aptamer exhibited a dissociation constant in the nanomolar range and did not bind with other structurally similar chemicals. Since then, a number of aptamers have been developed and integrated in assays for the detection of AFB1 [181], M1 [182], FB1 [183], ZEN [184], DON [185,186], PAT [187], T-2 toxin [188], and EAs [189]. The sequences and affinity of those commonly used mycotoxin aptamers are summarized in Table 4. The dissociation constants, which indicate the affinity of aptamers, are from nanomolar to micromolar ranges.

Compared with pAbs and mAbs, aptamers may be superior in terms of stability, size, and production. However, the commercialization of aptamer-based methods for mycotoxin detection is not as fast as expected. The only report on commercial aptamer-based products was from NeoVentures Biotechnologies, Inc. (London, ON, Canada) for purification and determination of AFs and OTA [193]. Thus, the application of aptamers for rapid determination of mycotoxins in different matrices should be further studied.

### 2.3. Short Peptides

**Figure 4:**
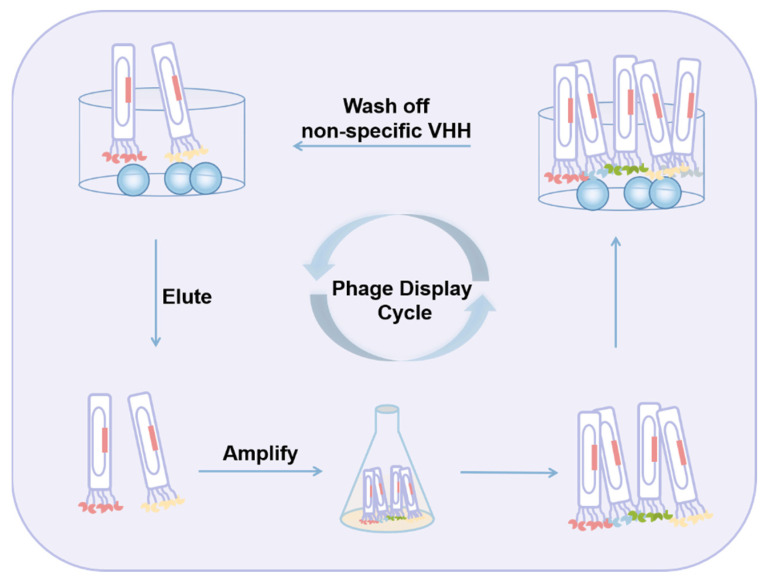
Specific peptide screening by phage display technology.

Molecular recognition by short peptides is a rapidly growing area of research. Peptides have regular structures and therefore can recognize functional groups on the targets through non-covalent interactions (e.g., electrostatic, hydrogen bonding, hydrophobic effects, and van der Waals forces). Advantages of peptide-based receptors are that they can be synthesized in vitro, easily modified and fused to other tags, and are less prone to activity loss under harsh conditions. Peptides with specific binding activity can be obtained by two different approaches, i.e., phage display and combinatorial synthesis. By phage display technology, peptides of a given length whose sequences are randomly generated are synthesized in vitro and expressed on the surface of bacteriophages. As shown in Figure 4, the phage library is incubated with the specific antigen immobilized on a microplate or magnetic beads. The unbound phages are washed away, and the bound phages are eluted and reinfected into bacteria for amplification. After several rounds of panning, peptides with high affinity to the antigen can be obtained. Theoretically, peptides with the ability to bind to a particular ligand can be selected if the library is large enough. In fact, with the limitation of library size and panning method, peptides with recognizing properties towards molecules, especially small molecules, are not easy to obtain. A number of phage-displayed peptides towards particular antibodies were developed and applied as mimotopes to replace the free toxins or their conjugates in immunoassays for mycotoxins [194,195,196,197,198,199,200]. However, obviously, there was no reported successful example of mycotoxin-specific peptides obtained by phage display method.

In contrast to phage display technology, which is based on panning of a large number of peptides that were randomly synthesized and displayed on phage particles, combinatorial peptides can be designed and synthesized on purpose based on the structure of mycotoxins. Tozzi et al. obtained tetrapeptides with binding properties towards AFs by a combinatorial approach, which was the first research report on peptides with binding ability towards mycotoxins [201]. The binding constants of selected peptides were in the range of only 8.3 × 10^3^ M^−1^ to 12.0 × 10^3^ M^−1^, and the selectivity was similar with that shown by a commercial antibody. Molecular modeling software (e.g., SYBYL) can be applied to facilitate the design and synthesis of peptide sequences [202]. By using computational modeling, they designed two peptide ligands for OTA. Both of the peptides exhibited a binding strength to OTA with K_D_ value in the micromolar range, i.e., 11 to 15.7 μM, which are similar with those obtained by combinational chemistry [203,204]. With the advantages of easy availability and low cost, however, specific peptides have been developed only towards OTA and AFB1 [205]. The application of specific peptides as recognition elements in determination of other mycotoxins are not available yet.

### 2.4. Molecularly Imprinted Polymers (MIPs)

**Figure 5:**
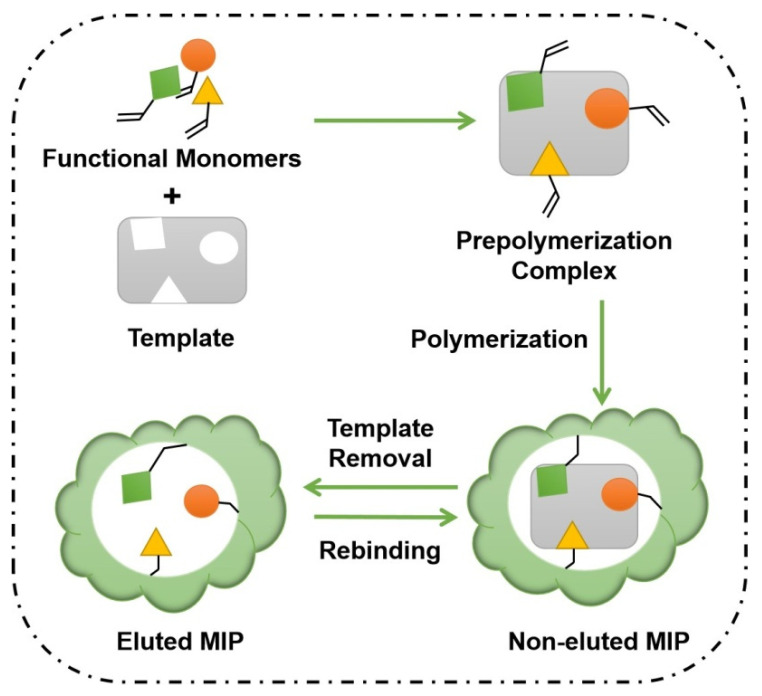
Synthesis procedure of specific MIP.

Other than the recognition elements mentioned above, molecularly imprinted polymers are not biological receptors but synthetic polymers. Thus, compared with other biorecognition elements, MIPs are more stable over varying conditions, such as temperature, pH value, and organic solvents, and easier to be produced at a relative lower cost and can be reused for several times [206]. MIPs are prepared by polymerization and crosslinking of functional monomers in the presence of the target molecule, called template, which is a catalyst and suitable porogen (Figure 5) [207]. After removing the original template, it results in a three-dimensional network that contains specific recognition cavities, which are complementary in shape and size with the target. These artificial materials thus can recognize a particular target molecule mimicking the biological activity of natural receptors.

To obtain MIPs with high affinity and selectivity, two highly important factors should be considered, i.e., template molecule and monomer selection. Generally, the template molecule plays a vital role in the development of MIPs with high affinity. As for mycotoxins, the template is harmful and expensive; thus, it is not feasible for many laboratories to manipulate with hundreds of milligrams of mycotoxins. As an alternative, a template can be used that mimics the structure of target compound as best as possible [208,209]. Baggiani et al. developed the first MIPs that recognize OTA, using N-(4-chloro-1-hydroxy-2-naphthoylamido)-(L)–phenylalanine as a mimic template [210]. The dummy template is stable, less toxic, and easy to prepare. It was found that the carboxyl, phenolic hydroxyl, and peculiar substructures are critical structures for OTA imprinting.

The selection of optimal functional monomers is the primary step for the preparation of MIPs. However, the large library of monomers and the complexity of interactions among template and monomers make the selection a big challenge [211]. To facilitate the design of proper monomers, computational modeling method can be employed [212]. Sergeyeva et al. synthesized nanostructured polymeric membranes as a recognition element and developed an MIP-based fluorescent sensor for AFB1 determination [213]. The selection of functional monomers was performed from a virtual library using computational modeling. By using ethyl-2-oxocyclopentanecarboxylate as a dummy template, AFB1 MIP membrane of high selectivity was synthesized. In another research [214], also using computational method, molecular interactions between FB1 and different acrylic monomers were analyzed, and an appropriate monomer was selected for MIPs development. NanoMIPs were produced with high specificity and successfully applied in an MIP-based immunosorbent assay in the replacement of the primary antibody. Furthermore, the properties of the non-imprinted polymer (NIP, blank polymer), which is synthesized in parallel without addition of the template, is crucial for the evaluation of specific binding ability of the MIPs. Maier et al. developed an MIP-based SPE column for the enrichment of OTA from red wine, followed by HPLC quantification [215]. However, the MIP-based SPE did not reveal as much superior to NIP; i.e., the retention of OTA depended mainly on non-specific binding to the polymeric material other than specific retention by the imprinted binding sites. Baggiani et al. observed that if NIPs had no affinity toward a target molecule, the corresponding MIPs would display poor imprinting efficiency [216]. On the other hand, if the NIP has good binding behavior, the imprinted polymer will show enhanced binding ability. They concluded that the obtained results are valid for a wide variety of MIPs. Several researches have been published about blank polymers that performed good binding ability and selectivity [217,218]. Compared with MIPs, the synthesis of blank polymers avoids the use of template, which is more environmentally friendly and less expensive. Furthermore, the slow release of template during storage is also eliminated. There are other factors that influence the property of MIPs, including polymerization temperature [219], solvent [220], and polymerization procedure [221], etc. The use of MIPs as receptor is becoming more common in analysis area due to its inherent thermal and chemical stability, ease of preparation, and low cost.

Most common application of MIPs is solid-phase extraction, the so-called MISPE (Molecularly Imprinted Solid Phase Extraction), for purification of the toxins prior to further analysis, for example, chromatographic assay [222]. MIPs as sorbents have been developed for purification of AFs [223,224], ochratoxins [225], FMs [226], CIT [227], ZEN [208], T-2 toxin [228], PAT [229], metergoline [72,230], and alternariol [231]. Compared with other selective sorbents, such as immunoaffinity columns, MISPE has several advantages: (a) MIPs can bear a high number of binding sites, whereas biological acceptors only have one or two. Thus, the capacity of MISPE is usually higher than that of IACs [232]. Lucci et al. developed a clean-up method employing MIP as selective sorbent for the preconcentration of ZEN [233]. The column had a capacity of no less than 6.6 μg, whereas the ZearalaTest immunoaffinity column from VICAM was saturated when loading 1.6 μg of ZEN. (b) MIPs demonstrate very good thermal and chemical robustness, leading to repeatable usage without loss of activity. Taking OTA determination as an example, the MISPE could be reused for at least five times with wine [234] and 14 times with beer [235] after regeneration. (c) Most affinity sorbents are made of binding elements immobilized on a solid support, such as agarose. The development of MISPE is more convenient. Once the MIP for a target is obtained, the selective MISPE can be developed by simply packing a small amount of imprinted polymer into a cartridge. Furthermore, MIPs can also act as an adsorbent to remove and control mycotoxins in foodstuff, such as the decontamination of milk by removing AFs [236] or removing PAT from apple juice [237]. MIPs have also been employed in the development of sensors for mycotoxin analysis, which will be discussed in the following section.

## 3. Areas of Application of Recognition Elements for Detection of Mycotoxins

### 3.1. Sample Preparation

Sample purification and clean-up is usually required in chromatographic analysis of mycotoxins given the complex matrices and trace amounts of targets in food samples. This step is crucial to get clean and concentrated extracts, therefore improving assays’ sensitivity to some extent. Owing to the high affinity and specificity of antibodies, immunoaffinity sorbents are powerful clean-up tools and are applicable in a wide range of food samples for single or multiple mycotoxin analysis [238,239,240,241]. Numerous immunoaffinity sorbents are commercially available worldwide (see Section 4) for the analysis of single or multiple mycotoxins. With similar properties, other molecular recognition elements have also been introduced as affinity sorbents, such as aptamer-based oligosorbents [242,243,244,245,246] and MISPE sorbents [232,247,248,249]. Sample purification technologies and their properties have been extensively discussed in previous publications [250,251,252] and therefore will not be covered in very much detail in this review. Rapid determination of mycotoxins should require only simple or no sample treatment; i.e., complicated clean-up steps should be avoided. There are also reports on immunoaffinity columns combined with immunoassays [253,254]. The most prevalent sample treatment in rapid analysis is liquid-liquid extraction (LLE). Mycotoxins are hydrophobic molecules, which are most dissolvable in organic solvents. Thus, they are usually extracted using polar solvents, such as methanol and acetonitrile. In conclusion, the presence of organic solvents in the extract requires relatively high stability and tolerance of the biorecognition molecule.

### 3.2. Microplate- and Tube-Based Assays

Microplates, also termed microtiter plates or multi-well plates, became essential tools in analytical chemistry. The commonly used microplate-based assay for mycotoxin analysis is the enzyme-linked immunosorbent assay (ELISA). There are two types of competitive ELISA formats used in mycotoxin determination, including direct (dcELISA) and indirect ELISA (icELISA). In direct ELISA, an unknown amount of mycotoxin in samples competes with analyte-enzyme conjugate for the coated anti-mycotoxin antibody, and the signal is then developed by adding the enzyme substrate. In indirect ELISA, analyte-protein conjugate (e.g., BSA, OVA, and KLH) is coated on the microplate, and competition for the limited amount of antibody takes place between the immobilized antigen and free analyte.

**Figure 6:**
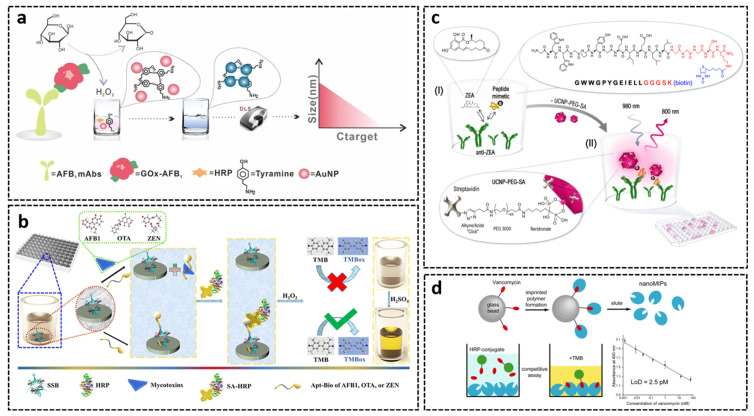
(**a**) Schematic illustration of DLS-dcELISA method combined with H_2_O_2_-mediated tyramine signal amplification system. (**b**) Scheme of green ELISA based on SSB-assisted aptamer. (**c**) Scheme of the competitive ULISA for the detection of ZEN. (**d**) Scheme of molecularly imprinted polymer nanoparticle-based assay for vancomycin determination. Reproduced with permission from [266,284,294,297].

The anti-mycotoxin antibody (primary antibody) can be labeled with an enzyme directly, or a secondary antibody enzyme conjugate is added for color development. The most commonly used enzyme is horseradish peroxidase (HRP), which catalyzes the oxidation of TMB by hydrogen peroxide and results in a blue color. Alkaline phosphatase (AP) is more stable and sensitive compared with HRP but with a higher cost. In addition to enzymes, different types of reporters have been developed for signal enhancing, such as polyHRP [255], fluorophores [256,257], functionalized magnetic beads [258,259,259,260,261,262,263], and upconverting luminescent nanoparticles [264]. In comparison to enzymes, these signal transducers usually have higher stability, enhanced signal, and lower price. Glucose oxidase (GOx), which can convert glucose by utilizing molecular oxygen to gluconic acid and hydrogen peroxide (H_2_O_2_), has also been employed as a reporter [265]. In the presence of HRP, H_2_O_2_ was converted to hydroxyl radicals and induced tyramine-mediated AuNP aggregation, thereby resulting in a dramatic change in visible color and dynamic light scattering (DLS) intensity (D_H_), which can be recorded with a DLS analyzer. By using this system, Zhan et al. developed a DLS-enhanced direct competitive ELISA for AFB1 detection in corn [266]. From Figure 6a, in the absence of AFB1, GOx-AFB1 was captured by anti-AFB1 mAb immobilized on the microplate, which could induce AuNPs aggregation in the presence of HRP and tyramine, with an intense D_H_ value. The LOD of the assay was 0.12 pg/mL, 153-fold lower than plasmonic ELISA and 385-fold lower than colorimetric dcELISA. QDs of variable size have different colors, thus facilitating the construction of microplate immunoassay for multiplex mycotoxin detection. Beloglazova et al. synthesized CdSe-based QDs with different emission spectrum and developed double-analyte multiplex assay (DAM) for simultaneous determination of ZEN and AFB1 [267]. In the DAM assay, two specific antibodies were immobilized in the same well of the microplate. Analytical signal was detected for both analytes by double scanning of the wells with different emission wavelength.

RAbs have been developed and applied in ELISA for AFB1 [268,269], OTA [270,271], ZEN [95,110], DON [171,272], FB1 [273,274], CIT [275], and T-2/HT-2 toxins [94]. One of the major advantages of the rAb-based ELISA is that rAbs-reporter fusions can be expressed directly based on genetic engineering, which eliminates the chemical synthesis of antibody-reporter or use of commercial secondary antibody. Various rAb-reporter fusions have been constructed, e.g., alkaline phosphatase (AP) [122], green fluorescent protein (GFP) [276], and HRP [277], which provide a valuable tool in the construction of microplate-based assays. Nanoluciferase (Nluc) is a novel luminescence tracer that offers excellent performance in immunoassays [278]. Wang’s group isolated specific Nbs against *Alternaria* mycotoxin tenuazonic acid and fused with Nluc by genetic engineering technique [125]. Based on the bifunctional fusion, a two-step bioluminescent enzyme immunoassay was constructed. The IC_50_ value of the assay was 8.6 ng/mL, which is six-fold more sensitive than ELISA.

Aptamers can also be applied as bioreceptor in microplate-based assays, named as enzyme-linked aptamer sorbent assay (ELASA). In direct format, the aptamer is coated on the microplate, and competition occurs between free analyte and analyte-reporter conjugate. The immobilization strategy of aptamers on the plate is of great importance to maintain its high binding affinity. Attachment of biotinylated aptamer on streptavidin/avidin modified microplate is the most commonly used procedure [279]. In indirect competitive ELASA, antigens or short, complementary DNA strands are coated on the plate, followed by addition of biotinylated aptamer and samples containing an unknown amount of mycotoxin for competition. Then, streptavidin-modified enzyme and substrate is added for color development. The aptamer can also be functionalized directly with a reporter, such as HRP [280], thrombin [281], and fluorescein [282,283], etc., which reduces the detection period and sometimes increases the assay’s sensitivity. By using single-stranded DNA-binding protein (SSB) as the competitive antigen and a specific aptamer as the bioreceptor, Xing et al. [284] constructed a novel green ELASA system for mycotoxin detection. As shown in Figure 6b, immobilized SSB and free targets compete for binding with the aptamer. SA-HRP was subsequently added for color development. This method was successfully applied for the analysis of AFB1, OTA, and ZEN in corn, with an LOD value of 112 ng/L, 319 ng/L, and 377 ng/L, respectively.

As stated already, only a few peptide receptors have been successfully designed and used as an alternative to antibodies in mycotoxin ELISA. By immobilization of anti-OTA peptide NFO4 on a microplate, Bazin et al. established a peptide-based dcELISA for OTA detection [204]. The assay could detect OTA up to 2 μg/L in red wine, which highlights the possibility of using a peptide as biorecognition element in immunoassays. On the other hand, peptides that serve as epitope mimics have been introduced as valuable substitutes for mycotoxin-protein conjugates in competitive immunoassays. Analyte-protein conjugates are usually involved in competitive immunoassays as competitive binders with the antibody. However, the synthesis of mycotoxin-protein conjugate can be difficult, time consuming, and even hazardous to users and the environment. Moreover, lot-to-lot variation and low conjugation efficiency make the synthesis of competing mycotoxin antigen one of the major challenges in developing immunoassays. Phage-displayed peptides (mimotopes) have been proposed as an alternative way to overcome these drawbacks [285,286]. Such mimotopes bind to the same antibody paratope as target toxin and thus can substitute hapten conjugates in an immunoassay. The ease of genetic engineering and low production cost make peptide mimotopes an attractive choice as antigen surrogates [287]. At present, a variety of peptide mimotopes have been identified and applied in the analysis of mycotoxins, including AFB1 [288], ZEN [289,290], OTA [291], FB1 [292], and DON [293]. Peltomaa et al. identified a ZEN-mimicking peptide by phage display and synthesized it with extended biotin sequence on C-terminus [294]. As can be seen in Figure 6c, anti-ZEN mAb was coated on the microplate, and peptide mimotope competed with free toxin in sample for limited antibody binding sites. Afterwards, streptavidin-conjugated upconversion nanoparticles were added to develop an upconversion luminescence signal for ZEN quantification. This dcULISA has an LOD of 20 pg/mL (63 pM) with high specificity towards ZEN.

By replacing primary antibody with MIPs, biomimetic or pseudo-ELISA have been proposed for mycotoxin determination. The attachment of MIPs on the microplate is a key step for the successful development of a biomimetic ELISA. Given the hydrophobicity property, coating of MIPs on the polystyrene microplate is rather complex. In one approach, polymers are grafted directly on the plate in the presence of template and form a molecularly imprinted film [295,296]. Chianella et al. developed a novel immobilization method by using MIP nanoparticles (nanoMIPs) [297]. As illustrated in Figure 6d, stable coating could be achieved by simply loading nanoMIPs into the microplate wells, followed by evaporation of the solution. This technique is simple and analogous to physical adsorption of antibody in ELISA [298]. By using this method, Munawar et al. proposed a nanoMIPs-based assay (MINA) for the determination of FB1 [214]. Competition between FB1 and HRP-FB1 conjugate for binding of immobilized nanoMIPs occurred, followed by colorimetric reaction with enzyme substrate. The optical density was then used for quantitative determination of FB1, which is analogous to ELISA. The assay was shown to be 22 times more sensitive compared to a mAb-based ELISA, with a limit of detection of 1.9 pM and a linear range of 10 pM–10 nM. The 53 maize samples were analyzed by MINA, and the results were good, correlating with those obtained using ELISA and HPLC [299]. In comparison with antibody-coated microplates, the major advantage of the MIP-coated plates is its high stability. They can be stored under high temperature for a prolonged time without affecting the sensitivity of the assay. This characteristic makes them applicable for cost-efficient, room-temperature storage and transportation.

### 3.3. Lateral Flow Assays

**Figure 7:**
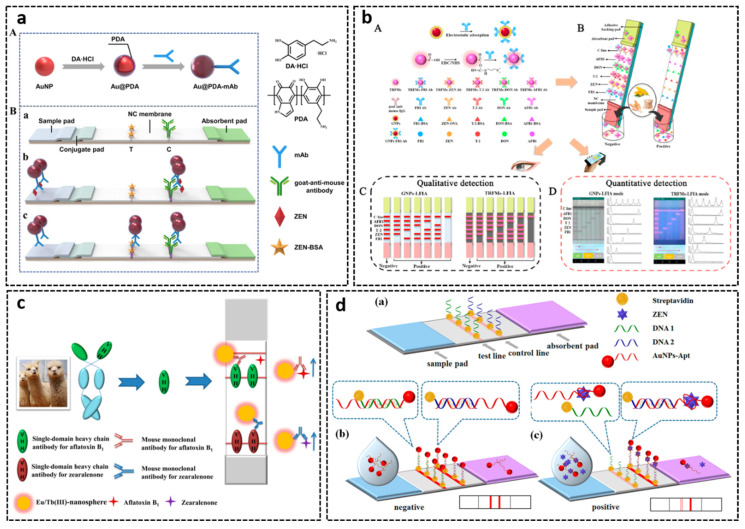
(**a**) Scheme of polydopamine-coated gold nanoparticles-based lateral flow immunoassay for ZEN detection. (**b**) Schematic diagram of smartphone-based GNPs and TRFMs-LFIAs for multiplex mycotoxins detection. (**c**) Schematic illustration of anti-idiotypic nanobody-based TRFICA for AFB1 and ZEN. (**d**) Scheme of aptamer-based lateral flow test strip for ZEN.Reproduced with permission from [310,322,323,329].

Lateral flow assay (LFA) is based on the movement of liquid sample along a strip of polymeric material, generally nitrocellulose (NC) membrane, for qualitative, semi-quantitative, and, to some extent, quantitative determination of analytes. Compared with microplate-based assays, LFA needs less detection period and is much easier to conduct; thus, it has been widely applied in point-of-care diagnostics and on-site monitoring. For the determination of small molecules like mycotoxins, LFA is based on a competitive format that is the same as microplate-based assays. Up to now, pAb- and mAb-based LFAs have been already developed for detecting mycotoxins, including AFB1 [300], AFM1 [301], DON [302], OTA [303], ZEN [304], FB1 [305], T-2 toxin [306], cyclopiazonic acid [307], and tenuazonic acid [308]. The assay is executed by adding small sample volume on the strip, allowing analytes of interest flow through the membrane. After a while, qualitative or semi-quantitative result is revealed by the appearance of a test line (T-line), and quantification can be realized by an optical reader. Given the benefits of strong red color, good stability, easy-of-synthesis, and low toxicity, gold nanoparticles are the most common labels in LFA [309]. To further enhance the color intensity of gold nanoparticles and facilitate the sensitivity of LFA, Xu et al. [310] synthesized polydopamine (PDA)-coated AuNPs as signal-amplification label for detection of ZEN in maize. PDA coating served as a linker of mAb and the nanoparticles. PDA-coated AuNPs was proven to be more stable and less easily aggregated with a stronger color brightness than that of AuNPs. From Figure 7a, in the absence of ZEN, a red band is observed due to the accumulation of Au@PDA-mAb on the T-line. Conversely, when there is an amount of ZEN in the sample, less or no recognition position is available to capture antigen on the T-line, which resulted in no line or a weaker line. The LOD of this assay is 7.4 pg/mL, which was 10-fold lower than that of AuNP-based LFA. In recent years, with the development of novel nanomaterials, extensive efforts have been devoted to increase the sensitivity of LFA for mycotoxin analysis by utilizing new labeled probes with stronger signal [311,312]. Therefore, different types of detection agents have been developed and applied in LFA for signal generation, including quantum dots (QDs) [306], upconverting nanoparticles (UCNPs) [313], magnetic nanoparticles [314], and near-infrared (NIR) fluorescent dye [315], etc. The analyte-antibody-probe complex continuously migrates by capillary action and competes for binding with the antigen deposited on the test line.

Due to the complexity of the co-occurrence of mycotoxins, the demands of simultaneous detection of multiple mycotoxins are increasing. To date, a number of multiplex LFAs for mycotoxins have been successfully developed with good performance, which could simultaneously detect up to six mycotoxins [315,316,317,318,319,320,321]. By using AuNPs and time-resolved fluorescent microspheres (TRFMs) as corresponding signal labels, two types of LFAs (AuNPs-LFA and TRFMs-LFA) were established by Z. Liu et al. for simultaneous detection of AFB1, ZEN, T-2, DON, and FB1 (Figure 7b) [322]. The visible LOD for the five mycotoxins were 10/2.5/1.0/10/0.5 μg/kg (AuNPs-LFA) and 2.5/0.5/0.5/2.5/0.5 μg/kg (TRFMs-LFA). By integration with a self-designed, smartphone-based, dual-mode device, quantification of the five mycotoxins was realized with LODs of 0.59/0.24/0.32/0.9/0.27 μg/kg and 0.42/0.10/0.05/0.75/0.04 μg/kg, respectively.

Although very few LFAs were introduced using rAbs as bioreceptors, the anti-idiotype nanobody (Aldnb) could serve as surrogate antigen on an immunochromatographic strip. Li’s group reported a time-resolved fluorescence lateral flow assay based on two anti-idiotypic nanobodies for simultaneous detection of AFB1 and ZEN in maize products [323]. As can be seen in Figure 7c, Aldnb were coated on NC membrane as test lines for AFB1 and ZEN, respectively. Anti-AFB1 mAb and anti-ZEN mAb were conjugated with Eu/Tb (III)-nanospheres as detector. For negative samples, the probes were captured by Aldnb immobilized on T-lines. For positive samples, target toxins in the samples reacted with mAb-probe, resulting in less or no probe captured on the T-lines. The intensity of T-line and C-line (control line) was measured by a homemade portable fluorescence spectrophotometer. A linear relationship between T/C values and logarithm of concentration of AFB1 and ZEN was constructed and applied for quantification.

In addition, peptide mimotopes, with the function of binding to the corresponding antibody, can also be used as antigen mimetics in LFA for mycotoxin analysis [324]. Yan et al. applied phage-displayed peptide and peptide-MBP (myelin basic peptide) fusion onto the T-line as the mimetic antigen. CdSe/ZnS QDs and QD-nanobeads with excellent optical property were conjugated with corresponding mAb as a signal reporter for rapid and simultaneous detection of FB1, ZEN, and OTA [325]. Under optimal conditions, the peptide-MBP-based LFA could detect 0.25 ng/mL FB1, 3.0 ng/mL ZEN, and 0.5 ng/mL OTA visually within 10 min.

Given the nature of nucleotide, aptamer can be hybridized with complementary DNA, and once the targets are present, the hybridization is deconstructed. Based on this property, aptamer-based LFAs have been designed for AFB1, ZEN, and OTA [326,327,328,329,330]. Wu et al. developed an aptamer-based lateral flow test strip for ZEN detection based on the competitive combination of aptamer with toxin and its complementary DNA (DNA 1) on the test line [329]. In this format, 3′-thiol- and poly A-modified OTA aptamer was synthesized and labeled with AuNPs. As shown in Figure 7d, in the absence of ZEN, AuNPs-Apt hybridizes with DNA 1 that is labeled with streptavidin and biotin-modified complementary DNA 2 that is immobilized on the T-line, causing a visible red line. If ZEN is present in the sample, AuNPs-Apt would bind with toxins, resulting in no line or a red line with weaker intensity. The more target analytes in the sample, the weaker intensity of the T-line. The control zone is loaded with biotin-modified polyT, which would hybridize with the polyA tail of the aptamer regardless of the presence of ZEN. The strip could detect ZEN in a range of 5–200 ng/mL, and the visual LOD was 20 ng/mL. Since a minimum of 500 s for hybridizing DNAs on microarray is usually required, it is difficult to obtain a strong and valid signal on the NC membrane by hybridization within 10 min [331]. To address this problem, Shim et al. developed an aptamer-based dipstick assay for AFB1 determination. In this approach, the biotin-modified aptamer was first incubated with sample solution and Cy5 dye-modified complementary DNA probe, which could assure adequate time for DNA hybridization [181]. Streptavidin and anti-Cy5 antibody were immobilized on test and control zone, respectively. The assay could be finished within 30 min with an LOD of 0.1 ng/mL for AFB1 in buffer.

Most of the reported aptamer-based strip assays are based on the competition binding of free toxin and complementary DNA with aptamers. One key factor that affects the reaction is the length of complementary DNA. If the length of complementary DNA is the same with aptamer, the latter would rather hybridize with complementary DNA than combine with the target. Taking advantage of the high affinity of aptamer-complementary strand and the binding efficiency of aptamer-target, Zhu et al. designed a dual-competitive LFA for AFB1 determination [332]. In this assay, AFB1-BSA was deposited on the test zone and competed for binding to aptamer with free toxin. In the presence of AFB1, the Cy5-labeled aptamer combines with toxin, which could not be captured by the immobilized antigen, resulting in a decrease in fluorescence signal on T-line. When the aptamer-AFB1 complex arrived at control zone, the aptamer hybridized with complementary DNA and dissociated with the toxin at the same time, owing to the higher affinity of hybridization. As a result, the higher the concentration of AFB1, the higher the intensity of the signal on the C-line. To increase the validity of the strip, the ST/SC ratio was employed for quantification of AFB1. The assay achieved an LOD of 0.1 ng/mL and a linear range of 0.1–1000 ng/mL.

Besides the benefits mentioned in the second section, aptamers can also show superiority over antibodies in the application on LFAs for mycotoxin determination. On one hand, aptamers can be synthesized with biotin, thiol, or fluorescent molecules for single-site conjugation, which facilitate quantitative analysis. Secondly, given the single-stranded DNA property, aptamer LFAs can be designed based on hybridization with complementary DNA, which eliminates the use of antigen. However, the limitation of this assay is hybridization deficiency, making it difficult to obtain a strong and reliable line on both test and control zones.

### 3.4. Biosensors

Biosensors are portable bioanalytical devices that incorporate biological recognition elements for binding of target molecules and a signal transducer to convert the biorecognition event into a measurable signal [333]. Based on various bioinspired recognition elements, mycotoxin sensors can be categorized into immunosensors [334], aptasensors [335,336], peptide-based sensors [205], and MIPs-based sensors [337,338]. Compared with other analytical methods mentioned above, it is much easier to realize real-time monitoring of reaction changes dynamically using biosensors, with output of the results in digital formats. Not only can the detection period be shorter, but the sensitivity, simplicity, robustness, and reusability can also be improved, making it possible to develop low-cost high-throughput screening methods for mycotoxins. A variety of transducers have been explored for mycotoxin sensor development. The electrochemical, optical, mass sensitive, calorimetric, and magnetic transducers stand out as the most important sensing platforms [339,340,341]. There is an increasing tendency for development of electrochemical immuno- and aptasensors [342,343,344].

**Figure 8:**
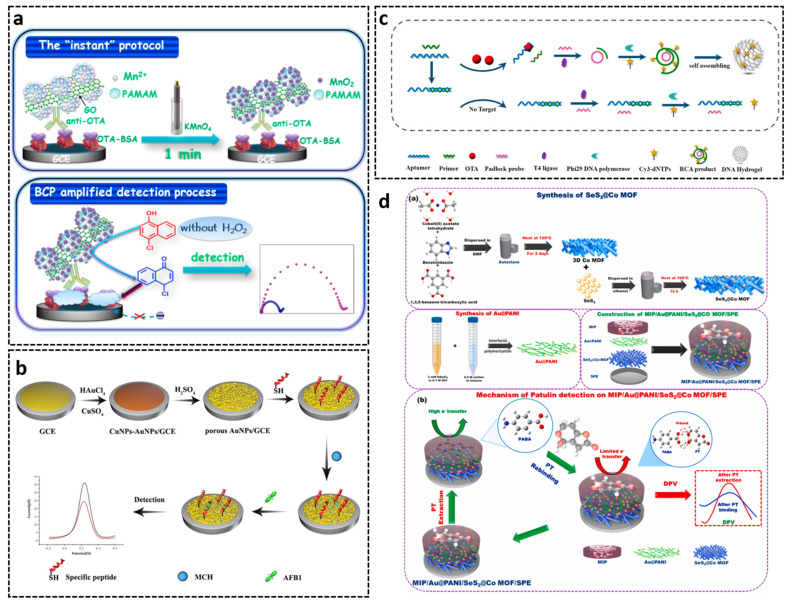
(**a**) Scheme of the amplified impedimetric immunosensor for OTA detection. (**b**) Scheme of electrochemical immunosensor for AFB1 detection based on specific peptide. (**c**) Scheme of fluorescent DNA hydrogel aptasensor for the detection of OTA. (**d**) Scheme of SeS_2_-loaded Co MOF with Au@PANI-comprised electroanalytical MIP-based sensor for PAT. Reproduced with permission from [205,345,364,399].

PAbs and mAbs are most commonly applied in the fabrication of mycotoxin immunosensors. J. Tang’s group developed an impedimetric immunosensor for OTA determination in red wine based on OTA-specific pAbs [345]. In this platform (Figure 8a), OTA-BSA was immobilized on the electrode. This conjugate competes with free OTA for graphene oxide nanosheets labeled anti-OTA pAb. This immunosensor exhibited an LOD of 0.055 pg/mL, with a working range between 0.1 pg/mL to 30 ng/mL. Zong et al. [346] developed a chemiluminescence immunosensor for AFB1, OTA, and CIT, using specific mAbs and glass-slide-immobilized antigens. A signal-on photoelectrochemical immunoassay for AFB1 based on enzymatic product-etching MnO_2_ nanosheets for dissociation of carbon dots was developed [347]. Under optimal conditions, the photocurrent increased with the increasing target AFB1 within a dynamic working range from 0.01 to 20 ng/mL with an LOD of 2.1 pg/mL. X. Tang et al. immobilized diacetoxyscirpenol-OVA on a microtiter strip and constructed a pressure-dependent immunosensor by labeling secondary antibody with Au@PtNP [348]. The mentioned immunosensors are based on indirect competitive immunoreactions, which require conjugating free toxin to a carrier protein or a signal probe as competitor. Peptide mimotopes, with the ability to bind to the same antibody paratope as the antigen, have been integrated into biosensor fabrication as a promising surrogate [293]. Hou et al. demonstrated an electrochemical immunosensor using phage-displayed peptide mimotope as the competing antigen for the detection of OTA [291]. In this case, the anti-OTA mAb was immobilized on a PEG-modified electrode. After competitive reaction of OTA and OTA-mimotope for binding to the anti-OTA mAb, the HRP-conjugated anti-M13 bacteriophage antibody was added to the sensor, and the quantification was realized by square-wave voltammetry measurement. The peptide-based immunosensor showed high selectivity and sensitivity, allowing the detection of OTA as low as 2.04 fg/mL in a linear range of 7.17–548.76 fg/mL. Label-free electrochemical biosensors have also been reported. For fabrication of an electrochemical immunosensor, Jiang et al. synthesized MoS_2_-thionin composites to modify the glassy carbon electrode (GCE), followed by coating of Pt-conjugated anti-ZEN mAb [349]. Square wave voltammetry (SWV) measurement was conducted to determine the concentration of ZEN. The peak current deceased with the increase of ZEN concentration. A linear range from 0.01 to 50 ng/mL and an LOD of 0.005 ng/mL was achieved by the electrochemical sensing platform. Recently, a label-free photoelectrochemical immunosensor based on antibody-immobilized photocatalyst g-C3N4/Au/WO3 was developed, which allowed the detection of AFB1 in the range form 1.0 pg/mL to 100 ng/mL [350].

Recombinant antibodies have great potential in biosensing systems. Z. Tang et al. developed a competitive FRET-based immunosensor by using QDs-labeled OTA and QDs-labeled nanobody as energy donor and acceptor, respectively [351]. Compared with traditional antibodies, the small size of Nb decreases the FRET distance between two QDs, making it more suitable for a sensitive FRET-based assay. The Nb-FRET immunosensor could detect OTA as low as 5 pg/mL within 5 min. A voltammetric immunosensor was constructed for AFB1 detection by X. Liu et al. [352]. The anti-AFB1 nanobody was coated on the surface of AuNPs/WS_2_/MWCNTs nanocomposites serving as recognition element and AFB1-streptavidin conjugate as competitor. This assay displayed a linear range from 0.5 pg/mL to 10 ng/mL with an LOD of 68 fg/mL. By using an anti-DON Fab fragment as recognition element, Romanazzo et al. [113] developed an Enzyme-Linked-Immunomagnetic-Electrochemical (ELIME) assay for DON detection in food samples. The sensor achieved a working range from 100 ng/mL to 4500 ng/mL and an EC50 of 380 ng/mL.

Specific peptides are also able to be applied in biosensors. Based on the crystal structure a AFB1-specific antibody, B. Liu et al. constructed a peptides library specific to AFB1 by using molecular docking and amino mutation [205]. The peptide P24 with highest affinity with AFB1 was selected and employed in an electrochemical immunosensor with signal enhancement of porous AuNPs. As shown in Figure 8b, P24 was immobilized on the surface of porous AuNPs/GCE electrode as a recognition element. The electrical current was detected by differential pulse voltammetry method for quantification of AFB1. The LOD of the assay was 9.4 × 10^−4^ μg/L with a linear range from 0.01 μg/L to 20 μg/L.

In the past decade, numerous aptasensors towards several mycotoxins have been introduced, including OTA [353], AFB1 [354], AFM1 [355], PAT [356], FB1 [357], ZEN, and T-2 toxin [358]. Based on their chemical nature, aptamers are more effective and robust under extreme pH and temperature conditions, making them attractive as reliable recognition elements in biosensors. Mycotoxin aptasensors mainly depend on the interactions between an aptamer and target toxin and its complementary strand or a signal probe, taking advantage of the unique property of nucleic acids, including configurational or conformational modifications under the formation of aptamer-target complex. Various detection modes have been applied, which can be mainly categorized into optical and electrochemical sensors. Optical methods, such as colorimetry [359], fluorescence [360], luminescence [361], FRET [362], and surface-enhanced Raman spectroscopy-based aptasensors [363], benefit from easy generation and provide high sensitivity. Based on self-assembly of rolling circle amplification (RCA), Hao et al. developed a fluorescent DNA hydrogel aptasensor for OTA [364]. As illustrated in Figure 8c, the OTA aptamer was first hybridized with the primer. In the presence of OTA, the aptamer tends to bind with the target, leading to the dissociation of primer. Free primer would combine with the padlock probe, which would initiate the RCA reaction, resulting in a formation of fluorescent DNA hydrogel. On the contrary, in the absence of OTA, no DNA hydrogel can be produced. The LOD of this aptasensor was 0.01 ng/mL, with a linear range from 0.05 to 100 ng/mL. In a similar approach, Abnous et al. designed a colorimetric aptasensor for AFM1 in milk based on the combination of CRISPR-Cas12a, RCA, and catalytic activity of gold nanoparticles [365]. The sensing method achieved an LOD of 0.05 ng/L, with a detection range from 0.2 to 300 ng/L. In comparison, electrochemical aptasensors are more cost-effective and feasible for on-site application owing to more simple instrumentation and fewer reagents [366,367,368,369]. This sensing mode mainly depends on the detection of changes of electric current occurring on electrode surface produced by recognition reaction.

Despite the binding affinity of aptamers, several factors should be considered to construct an aptasensor with high sensitivity. One is the aptamer’s immobilization strategy. The fabrication of an electrochemical aptasensor requires the immobilization of aptamer on an electrode, which could remarkably affect the binding activity of aptamers. To increase the immobilization efficiency, various efforts have been made to modify the sensing platform. For example, carbon quantum dots/octahedral Cu_2_O nanocomposite has been used to modify the glass carbon electrode and combine with aptamer through amino-carboxylic interaction [370]. The sensing platform allowed the detection of AFB1 with an LOD of 0.9 ± 0.04 ag/mL and a dynamic range from 3 ag/mL to 1.9 μg/mL. In addition, chitosan-functionalized acetylene black and multiwalled carbon nanotubes (CS@AB-MWCNTs) nanocomposite, with large specific surface area, good conductivity, and film-forming property, has also been proved to improve the immobilization of aptamer on electrode, thus increasing the detection sensitivity [371]. The other factor affecting the performance of an aptasensor is the signal amplification method. Many functional nanomaterials with outstanding physicochemical properties provide a powerful tool to improve the sensitivity of the developed electrochemical sensors. By using upconversion nanoparticles-doped Bi_2_S_3_ nanorods as photoactive materials, Gao et al. constructed a near-infrared light-induced photofuel cell-based aptasensor, allowing the detection for AFB1 in the range of 0.01–100 ng/mL, with an LOD of 7.9 pg/mL [372]. DNA amplification methods, including polymerase chain reaction (PCR) [373,374], hybridization chain reaction (HCR) [375,376], rolling circle amplification (RCA) [377,378], strand displacement amplification (SDA) [379,380], toehold-mediated strand displacement amplification (TMSD) [381], catalytic hairpin assembly (CHA) [382], and DNA machines, have also been applied in aptasensor construction to enhance the sensitivity [383]. Taking advantage of HCR, DNA walkers, and the properties of MoO_x_ nanomaterials, Wang and coworkers demonstrated an aptasensor for determination of OTA [384]. The sensitivity was greatly improved, with a detection limit as low as 3.3 fg/mL.

MIPs have received extensive attention for electrochemical sensors construction due to their unique advantages, such as high intrinsic stability and ease of preparation [385]. MIP-based electrochemical sensors have been utilized to detect mycotoxins, including AFB1 [386], OTA [387,388,389], DON [390], ZEN [391], FB1 [392,393], CIT [394], PAT [395,396,397], and T-2 toxin [398]. To obtain an ideal MIP-based electrochemical sensor with high sensitivity, the fabrication of the MIP on the electrode surface as well as the electrode modification strategy must be considered. Numerous methods have been utilized in the fabrication of MIPs, including electropolymerization, bulk polymerization, and precipitation polymerization, and among them, electropolymerization is a convenient way to prepare MIP membranes on the surface of the electrode given its rapid preparation, easy control of film thickness, and improved cohesiveness. Selvam et al. constructed an MIP-based disposable sensor for PAT [399]. In this strategy (Figure 8d), SeS_2_-loaded Co MOF was synthesized via a tangible hydrothermal technology and loaded on a screen-printed electrode surface to improve the conductivity and stability. Then, Au@PANI (gold polyaniline) nanocomposite was prepared and loaded on the MOF screen-printed electrode to achieve higher sensitivity. Finally, the MIP sensor was fabricated on the Au@PANI/SeS2@Co MOF-modified screen-printed electrode platform via electropolymerization. An electron-blocking layer was formed when PAT was captured by the imprinted cavities, which caused a decrease in the electrochemical signal. This sensor possessed excellent performance, with an LOD of 0.66 pM for PAT and a logarithmic linear range from 0.001 to 100 nM. By using a similar approach, Huang et al. constructed an MIP-based electrochemical sensing platform for PAT determination by electropolymerization [395]. The combination of thionine, PtNP, and nitrogen-doped graphene (NGE) was used to modify the glassy carbon electrode to enhance the electric signal. The LOD of the fabricated sensor was 0.001 ng/mL in the PAT concentration range of 0.002–2 ng/mL. In another study, an MIP sensor for DON detection was developed by preparation of an MIP membrane on COOH-MWCNTs-modified electrode surface via electropolymerization [390]. The sensor displayed effective surface area, good conductivity, high selectivity, and a good response towards DON, with an LOD of 0.07 μM in wheat flour samples.

To summarize, electrochemical biosensors are the most prominent among mycotoxin sensors owing to their sensitivity, low cost, and miniaturization. The quantification for mycotoxins is based on the interaction between analytes and recognition elements, which is transformed to electrical signals using amperometric, potentiometric, conductimetric, and impedimetric measurements.

## 4. Commercial Biorecognition Elements, Test Kits, and Analysis Systems for Mycotoxins Detection

Up to date, there is a high number of commercial recognition elements and test kits for mycotoxin analysis available on the market, and most of them are conventional antibodies and antibody-based test kits, including pAbs and mAbs, ELISA test kits, lateral flow assays, and immunoaffinity columns. In this section, we give a general overview on currently available commercial products and important worldwide suppliers. The collection does not claim to be complete. Besides direct marketing by manufacturers, distribution occurs mainly by regional retailers or specialized dot-com companies. The latter organize a direct contact between the product manufacturer and the customer; i.e., they act as an agent only [400].

### 4.1. Mycotoxin Antibodies

Mouse monoclonal mycotoxin antibodies, i.e., isotype IgG, are predominant on the market. However, polyclonal ones are still offered. To date, alternative biorecognition elements, with the exception of rAbs, are not commercially available as single products, i.e., not being part of a test kit (e.g., MIP-solid phase extraction columns). Some rAbs for mycotoxins were offered recently by Creative Biolabs (www.creative-biolabs.com accessed on 10 December 2021). Beside bioequivalent reagents (full-size IgG) with the identical primary sequence (for ZEN, OTA), scFv and Fab (for ZEN, OTA, AFM1) and also VHH single-domain antibodies (for 15-AcDON) are obtainable. Unfortunately, web-based product information documents are not complete. For example, data for cross-reactivity with metabolites and other mycotoxins are missing. Furthermore, if disposable, application notes should be made available for download to interested users.

As listed in Table 5, mAb and/or pAb are commercially available towards common mycotoxins. There are only very few suppliers of antibodies against PAT, T-2/HT-2, and EAs. A mAb-based ELISA kit against CIT can be obtained from Creative Diagnostics (www.creative-diagnostics.com accessed on 17 January 2022) (not listed in Table 5). Generally, important information, such as used immunogen, host species, purity, reactivity, advice for reconstitution and storage, recommended use, etc., are outlined in the disclosed data sheet. These details are essential to create your own immunoassay.

### 4.2. Microplate or Tube ELISA Test Kits

All commercial test kits are based on mAbs and pAbs. Products with use of alternative biorecognition elements are unknown. ELISA is commonly used in mycotoxin detection with the advantages of high throughput, sensitivity, and accuracy. Both direct and indirect immunoassay formats have been involved in commercial ELISA kits. The microplate is precoated with antibody (direct format) or mycotoxin-protein conjugate (indirect format) and blocked with protein. In direct format, mycotoxin standards or samples are then added to each well with conjugated mycotoxin-enzyme. In indirect format, standard solution or samples are added together with specific antibody and enzyme-conjugated secondary antibody. To achieve a fast detection, the incubation period (15–20 min) is usually less than that reported in research articles (30–60 min). The whole detection can be finished mostly within 30 min. Various signal readout techniques (e.g., colorimetry, fluorescence, chemiluminescence) in ELISAs have been reported. Aokin rapid analysis systems (www.aokin.com accessed on 10 December 2021) commercialized a hand-held and portable instrument (Aokin mycontrol analyzer FP) based on highly sensitive, patented kinetic fluorescence polarization technology, together with a set of detection kits (mycontrol kits, incl. SPE cleanup columns) for most of regulated mycotoxins to allow rapid and quantitative determination in food and feedstuffs on-site. Altogether, most commercial ELISA kits predominantly use colorimetry (note: In Table 5, the signal technique is not specified for individual ELISA kits.). The mycotoxin ELISA kits can be applied to food (e.g., milk), agricultural commodities (e.g., wheat, rice, maize, etc.), and feed products after a simple extraction procedure. Generally, the applications are accurately specified in the instruction manuals.

### 4.3. Lateral Flow Assays

Given the benefits of low costs, user-friendliness, rapidness (usually <15 min), little interferences, portability, long shelf life, and operation by nonspecialized personnel on-site, lateral flow assays have attracted considerable interest in food-safety area. The goal is to accommodate all the reagents required for a quantifiable test on a simple membrane, strip, or capillary. It should be possible to place a volume of sample on the carrier or dip it into the liquid sample (e.g., sample extract) and to determine the presence of target analyte from resulting depth of color or length of colored band. Depending on the kind of label, evaluation can be done by naked eye (visual inspection), or spots can be read out by an electronic device, e.g., a smartphone. Labeling of antibodies by nanogold, which leads to red lined for colorimetric evaluation, is dominant. Most commercially available test strips for mycotoxins are encased individually in a plastic container. With few exceptions (PAT, CIT, EAs), related tests are offered for all regulated mycotoxins (Table 5). Generally, these tests are used for qualitative and semi-quantitative determinations, especially for sample screening regarding compliance/exceedance of limit values and maximum residue limits (MRLs). So far, quantitative tests are less widely available. Analogous to ELISA formats, different test configurations are possible. Owing to the small size of the targets, common principle of LFDs for mycotoxins is the indirect competitive immunoassay. The LFD is a combination of thin-layer chromatography, i.e., diffusion over a distinct distance on a membrane, and detection of a specifically labeled immune-reactant. The main elements are a sample pad (for addition of liquid), conjugate pad (with adsorbed, labeled target antibody probe, i.e., the primary antibody), nitrocellulose membrane with fixed test line (T-line, with adsorbed analyte protein conjugate) and control line (C-line, with adsorbed secondary antibody specific for the primary antibody; this line serves as an indispensable confirmation that the test worked correctly), and absorption pad (for absorption of diffused liquid), all of which are generally fixed on a plastic carrier. Briefly, after the addition of sample extract to the strip, any mycotoxin present in the sample will bind to the labeled antibody probe and migrate together with unbound antibody along the membrane caused by capillary forces. The mycotoxin-protein conjugate on the T-line competes with free toxin in the sample extract for binding to the labeled antibody. If a visible line appears on the test zone, the concentration of mycotoxin is less than the cut-off level, which is a negative sample. Conversely, a positive sample results in no visible line on the test zone. Quantification of the target can be realized by a commercial strip reader, based on the intensity of T-line or T/C signal ratio. With the aim to obtain even more sensitive assays, new labels are used, for example, fluorescent nanoparticles, such as quantum dots (Shandong Lvdu Bio-Sciences & Technology Co., LTD., Binzhou, China) and Lanthanide chelates (Beijing WDWK Biotechnology Co., LTD., Beijing, China). So far, rAbs-, aptamers-, and MIP-based LFDs are not commercialized.

### 4.4. Immunoaffinity Columns

Immunoaffinity chromatography (IAC; sometimes also termed immunoextraction, IE) is a special kind of solid-phase extraction (SPE) and combines immunological and traditional methods. It has been widely used as mycotoxin clean-up prior to TLC, HPLC, GC, or LC-MSMS. It is focused on the time- and cost-saving removal of sample interferences and the selective preconcentration of the trace target(s) in front of the chromatographic analysis. The technique uses cartridges or columns made from plastic material or glass and filled with anti-mycotoxin-loaded sorbent (termed immunoaffinity support or immunosorbent). The method can be performed off-line or on-line depending on the available support material. High-performance supports, which are needed for on-line techniques, must be rigid and robust, mechanically stable, and perfusive. The common principle, after addition of sample extract, is to start with a washing step to remove unbound and weakly bound sample constituents. This is followed by desorption of specifically trapped target analyte(s) with a suitable eluent and, finally, injection into the chromatographic system. Except PAT and EAs, IAC columns are offered for all regulated mycotoxins (Table 5). Users should perform the procedures as described in test instructions of suppliers. Generally, the tests are offered for single use. Increasingly, IAC columns for the simultaneous enrichment of multiple mycotoxins are coming to the market. The current examples for mention include Myco6in1+ columns from VICAM (www.vicam.com accessed on 10 December 2021) and 11 + Myco MS-Prep^®^ from R-Biopharm (https://r-biopharm.com accessed on 10 December 2021) for simultaneous determination of AFs, OTA, FMs, DON, ZEN, and T-2/HT-2 in combination with LC-MS/MS. (note: Columns from VICAM also can detect nivalenol). To the best of our knowledge, commercial affinity columns for sample preparation based on rAbs and aptamers are not on the market yet. In contrast, MIP-based columns are already available. The company AFFINISEP (www.affinisep.com accessed on 10 December 2021) offers a set of cartridges (AFFINIMIP^®^ SPE) designed for the analysis of one specific family of mycotoxins (AFs, FMs, DON, ZEN, OTA, PAT) and a multi-mycotoxin cartridge for simultaneous extraction of mentioned families plus T-2/HT-2 in combination with LC-MS/MS. Further, an MIP clean-up column (EASIMIP™ Patulin) is available from R-Biopharm.

### 4.5. Multiplexed Analysis Platforms

The ability to test multiple mycotoxins simultaneously, termed multiplexing, has several advantages over traditional single-analyte testing and has gained increasing interest over the past decade. As presented previously, new rapid tests were developed to address this challenge. Furthermore, there is an increasing need to provide cost-efficient, rapid, and fully automated methods for routine analytical laboratories. One option is to integrate a set of single devices on a modular platform and control the complete analysis by suitable software. Examples, such as Cobas Analyzer from Roche Diagnostics (www.roche.com accessed on 10 December 2021) and ADVIA Centauer XP Immunoassay System from Siemens Healthineers (www.siemens-healthineers.com accessed on 10 December 2021), can be found in high-throughput laboratories, e.g., clinical in-vitro diagnostics and pharma screening. Related investments are only affordable by big players in the food-safety testing market.

Therefore, another direction of research is focused on the development of continuous devices, e.g., flow injection analysis (FIA), and new miniaturized platforms for multiplexed high-throughput analysis. The latter can be separated into two technologies. The first is particle-based methods (bead-based arrays), which use a set of differently labeled micro- or nanoparticles (also termed microspheres or beads) as carriers for positioning of detection reagents, and tests are performed in suspension (suspension arrays) [401]. The second is planar biochips (also termed microarrays, lab-on-the-chip), with use of site-specific positioning of the detection reagents on a microchip, encased in a microfluidic cassette [402]. The strengths of suspension arrays are high array density, high-throughput capacity, and opportunity for configuration of a multiplex assay on demand (customizable, i.e., distinct sets of microspheres). Both types of assays are strongly dependent on the availability of special reagents and materials, i.e., appropriately functionalized carriers (particles or microchips), dispenser (arrayer for spotting of chips), biochip reader or scanner for microarrays, and flow cytometer for suspension arrays, including evaluation software. Because of the complexity of food samples, the maximum number of cycles of determination/regeneration (operating life) with one and the same biorecognition surface is limited, and the trend goes in the direction of single-use materials (beads and biochips). The latter is also caused by the steadily more efficient and cost-effective production (Table 6).

The number of commercial biosensors that can detect the interaction of receptors with their targets in a preferably label-free manner and on time is steadily growing (Table 7). Important detection techniques are surface plasmon resonance (SPR) [403,404], quartz crystal microbalance (QCM) [405,406], microcantilever arrays [407], biolayer interferometry (BLI) [408], and electroswitchable biosurfaces (ESB) [409,410].

## 5. Conclusions and Future Perspective

Rapid determination methods based on biorecognition elements have been presented as promising tools for monitoring of mycotoxin contamination in food samples, which is a powerful supplement to highly sophisticated instrumental methods. In this review, the basic characteristics and application potential of commonly used recognition elements, including traditional pAbs and mAbs, and upcoming new receptors, such as rAbs, aptamers, peptides, as well as MIPs, were presented. Tremendous efforts have been dedicated over the last decade to develop receptors with further enhanced specificity, binding affinity, stability, and lower cost via improved antigen design, optimized screening strategies, expression or synthesis methods, and integration of new computational modeling approaches [411,412,413]. Consequentially, massive progress in the application of new receptors in various analytical formats, including microplate- or tube-based assays, lateral flow assays, solid-phase affinity support materials, and biosensors, have followed mainly in the academic sector. The product market on the global scale, however, clearly lags behind in bringing the new reagents, test kits, and technologies to the customers.

Disregarding some limitations, such as long production period and high costs associated with conventionally used pAbs and mAbs, they still dominate the field, both as available purified biorecognition reagents, receptors used in test kits, and affine binders of sample clean-up materials. The market for these reagents and tests is a competitive one, with global trading. It can be quite difficult and cumbersome to the customer to identify the original source of the antibody, and it often happens that, e.g., a mAb-producing cell clone was licensed to several companies for marketing either as purified antibody reagent or being part of a test-kit, branded or distributed under different names. However, there is a continuous demand for more cost-effective mycotoxin receptors with customized selectivity, affinity, and stability, i.e., engineered to fit the needs of the final application. From the current point of view, rAbs, especially the nanobodies, could be the most promising solution owing to progress of related technology. Furthermore, the availability and use of synthetic and naïve antibody libraries, which are rather large nowadays, could lead to the renouncing of obsolescent animal immunization. It is worth mentioning, for example, the EU directive 2010/63/EU on the protection of animals used for scientific purposes [414]. Creditably, Creative-Biolabs first made rAbs for OTA, ZEN, AFM1, and 15-AcDON commercially available.

**Figure 9:**
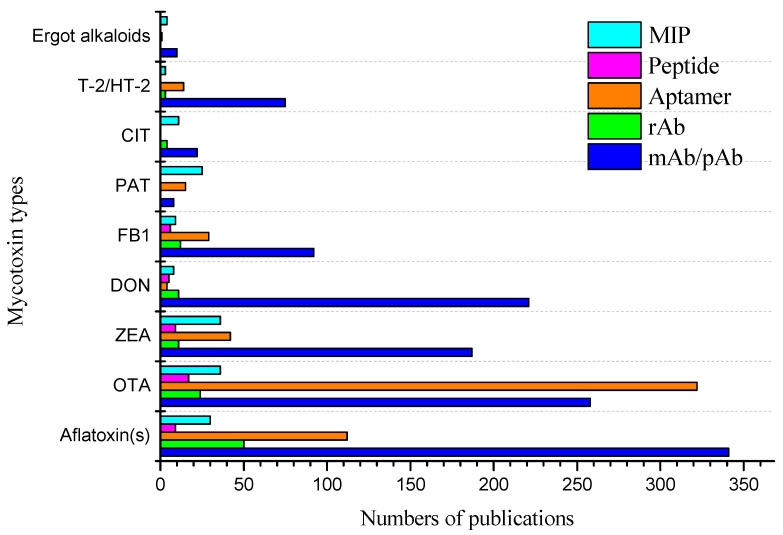
Numbers of publications on mycotoxins determination based on various recognition elements. Data were obtained in Web of Science until 3 December 2021.

The number of publications on the determination of mycotoxins based on various receptors was established and is presented in Figure 9. As can be seen, researches based on antibodies for mycotoxins detection are predominant. Aptamers are capable of recognizing targets with similar or even higher affinity compared to antibodies, with appealing characteristics in the aspects of production, stability, and signal labeling. However, it is still a challenge to obtain aptamers towards small molecules with desired characteristics. Peptide receptors can be obtained by phage-display technique or combinatorial synthesis, which are less prone to denaturation under high temperature and organic solvent. Nevertheless, mycotoxin-specific peptides and their applications are rarely reported except for OTA and AFB1. Yet, the application of peptide mimotopes as antigen substitutes might be a promising aspect in the future. Among all the receptors, MIPs are the most easily obtained, with increased thermal and mechanical stability. As biomimetic recognition materials, MIPs have attractive features mainly in the application to sample preparation. However, their affinity and specificity are generally lower compared to the other binding receptors. In future research, a variety of limitations, including but not limit to template leakage, non-specific binding, and low affinity, should be addressed.

Up to date, detection methods towards a variety of fungi metabolites with high toxicity and widespread occurrence have been extensively studied. In addition to common mycotoxins, for which the maximum permitted levels in food and feed products are regulated, those without a regulation also pose great harmfulness to humans and livestock, e.g., ergot alkaloid, citreoviridin, and sterigmatocystin, etc. However, studies on toxicology, risk assessment, and detection methods towards those emerging mycotoxins are still limited. Thus, there is an ongoing demand for the development of recognition elements, assays, and rapid test-kits for new mycotoxins, e.g., NX-toxins, degradation products (e.g., cis-ZEN), as well as unregulated but important mycotoxins (e.g., the enniatins).

To summarize, there is still a great deal of room for and challenges associated with advancements in recognition elements-related assays and biosensor development, which will move their application from laboratory to market. Especially, it can be expected that novel, customized recognition elements, such as nanobodies, aptamers, and MIPs, and rapid test kits based on these receptors might be increasingly available to users in the future. We suppose biosensors that allow label-free, multi-analyte determination will be found mainly in food and feed laboratories. In addition, new, cost-effective, and portable devices for rapid on-site analysis will be increasingly available. Finally, the crucial factors for the selection of the most appropriate method can be seen in regulatory issues, the objective of the analytical determination, sample type, facility of the laboratory, and, not to be overlooked, the experience of the analytical staff.

## Figures and Tables

**Table 1:** Summary of major mycotoxins and their characteristics.

Mycotoxins	Structure	Main Fungi Species	Commodities Affected	Toxic Effects
Aflatoxins: B_1_, B_2_, G_1_, G_2_, M_1_^*^	AFB_1_ 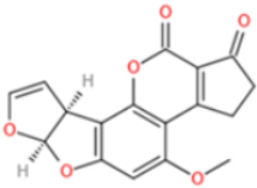	*Asp. flavus*, *Asp. parasiticus*	Nuts, spices, grains such as maize, rice, wheat, * milk and milk products, etc.	Carcinogenic, teratogenic, mutagenic, immunosuppressive [39]
DON	DON 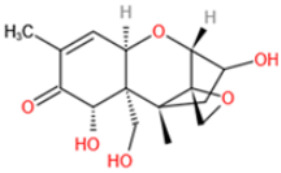	*F. graminearum*, *F. culmorum*, *F. cerealis*	Cereals, cereal products	Diarrhea, vomiting, anorexia, immune dysregulation [40]
ZEN	*trans*-ZEN 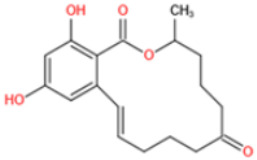 *cis*-ZEN 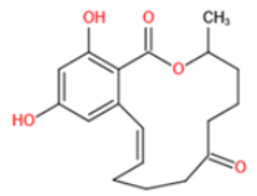	*F. graminearum (Gibberella zeae)*, *F. culmorum*, *F. cerealis*, *F. equiseti*, *F. crookwellense*, *F. semitectum*	Cereals, cereal products, maize, rice, beer, etc.	Hyperoestrogenic, hepatotoxic, haematotoxic, immunotoxic, genotoxic [41]
OTA	OTA 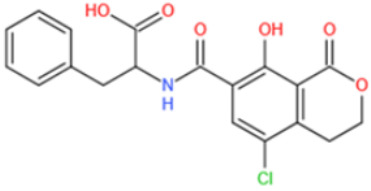	*Asp. ochraceus, Asp. niger,* *Asp. carbonarius,* *Asp. terreus,* *P. verrucosum,* *P. nordicum*	Cereals, wine, coffee, cocoa, beans, dried fruits, nuts, spices, cheese, etc.	Nephrotoxic, hepatotoxic, neurotoxic, teratogenic, immunotoxic [42]
Fumonisins: FB_1_, FB_2_, FB_3_	FB_1_ 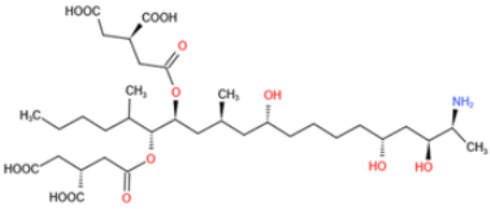	*F. verticillioides, F. proliferatum*	Mainly maize and maize-based products, sorghum, asparagus	Carcinogenic, cytotoxic, nephrotoxic, hepatotoxic [43,44]
T-2/HT-2 toxin	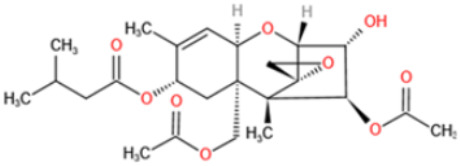 T-2 toxin 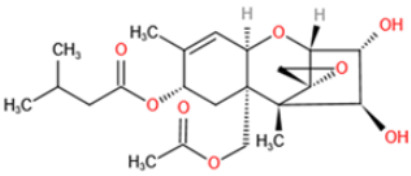 HT-2 toxin	*F. langsethiae, F. poae, F. sporotrichioides*	Wheat, rye, maize, soybeans	Growth retardation, myelotoxic, hemotoxic, necrotic lesions on contact sites [45]
PAT	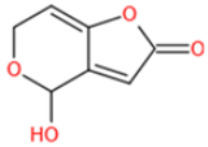	*P. expansum*	Fruits and vegetables	Nausea, vomiting and other gastro- intestinal symptoms, kidney damage [46]
CIT	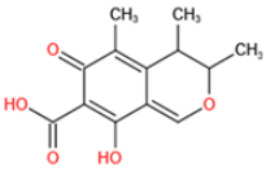	*P. citrinum,* *P. camemberti,* *Asp. terreus,* *Asp. niveus*	Fermented maize, cheese, corn, wheat, barley, red yeast rice, apples, brewed beer, cereal products	Nephrotoxic, may cause liver and kidney diseases, nervous system damage [47]

*AFM1 is only relevant to milk and milk products.

**Table 2:** Maximum permitted levels of mycotoxins in food according to regulations by China, European Union (EU) ^1^, and United States (U.S.).

Mycotoxins	Country	Maximum Permitted Level (μg/kg)
AFs	China	5–20 (0.5) *, (AFB1)
	EU	2–12 (0.1) *, (AFB1), 4–15, (sum of B1, B2, G1, G2),
	U.S.	20, (sum of B1, B2, G1, G2),
AFM1	China	0.5
	EU	0.05 (0.025) *
	U.S.	0.5
ZEN	China	60
	EU	50–400 (20) *
	U.S.	not set
OTA	China	2–10
	EU	2–80 (0.5) *
	U.S.	not set
DON	China	1000
	EU	500–1750, (200) *
	U.S.	1000
PAT	China	50
	EU	25–50, (10) *
	U.S.	50
FMs	China	in preparation
	EU	800–4000, (200) *, (FB1, FB2)
T-2/HT-2	U.S.	2000–4000, (FB1, FB2, FB3)
	China	not set
	EU	in preparation ^2^
CIT	EU	2000
EAs	EU	100–500, (20) *, (sum of 12 compounds)

^1^ Regulations (EC) Nos. 2002/32/EC, 1881/2006, 2021/1399); ^2^ 2013/165/EU: Commission. Recommendation; * Number in brackets refers to infant food and young children.

**Table 3:** Typical mycotoxin immunogens and obtained antibody characteristics.

Mycotoxin(s)	Immunogen Structure	Coupling Method	Antibody Type	Titer	IC_50_	LOD	Reference
Total AFs	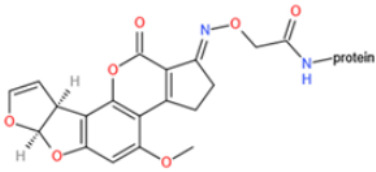 AFB1-oxime-BSA	Carbodiimide method	pAb	Higher than 1000	AFB1 1.8 ng/mL AFB2 16 ng/mL AFG1 20 ng/mL AFG2 320 ng/mL	AFB1 0.4 ng/mL	[160]
AFB1	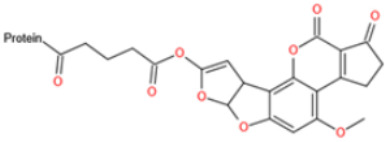 AFB2a-HG-BSA ^1^	Mixed anhydride method	pAb	710–800	0.15 ng/assay	0.02 ng/assay	[137]
AFM1	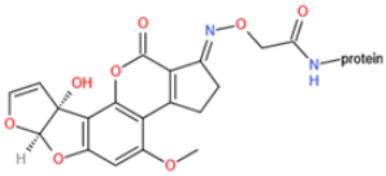 AFM1-BSA	Carbodiimide method	pAb and mAb	n.a.	25 ng/mL (mAb); 0.5 ng/mL (pAb)	n.a.	[82]
OTA	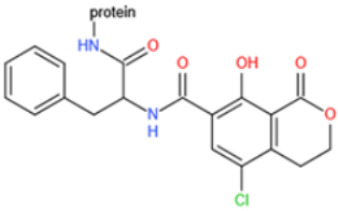 OTA-BSA	Carbodiimide method	pAb	n.a.	3 ng/mL	1 ng/mL	[161]
DON	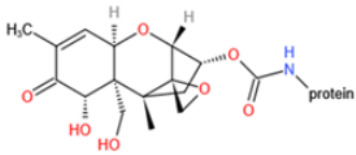 DON-BSA	N,N′-carbonyldiimidazole method	mAb	n.a.	9.84 ng/mL	n.a.	[162]
T-2 toxin	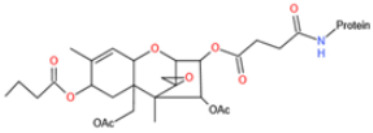 T-2-HS-BSA ^2^	Carbodiimide method	pAb	303	3.5 ng/assay	1 ng/assay	[146]
ZEN	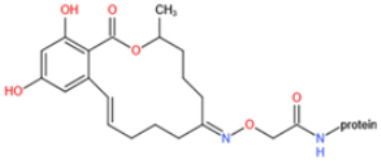 ZEN-oxime-BSA	Mixed anhydride procedure	pAb	5120	n.a.	0.5 ng/mL	[149]
	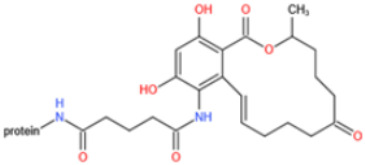 5-NH_2_-ZEN-BSA	Glutaraldehyde method	mAb	520	11.2 ng/mL	0.3 ng/mL	[153]
ZEN	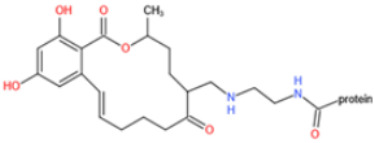	Mannich reaction	pAb and mAb	30,000	233.35 ng/mL (pAb); 55.72 ng/mL (mAb)	n.a.	[154]
	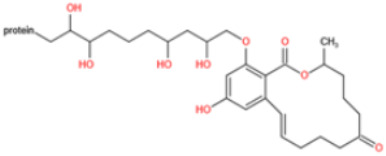	1,4-Butanediol diglycidyl ether method	mAb	1.024 × 10^6^	1.115 ng/mL	n.a.	[155]
FB1	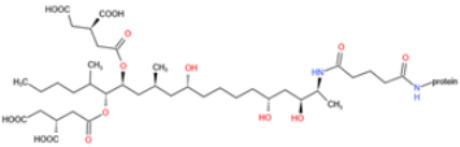 FB1-KLH	Glutaraldehyde method	pAb	10,000	0.45 ng/mL	0.1 ng/mL	[163]
CIT	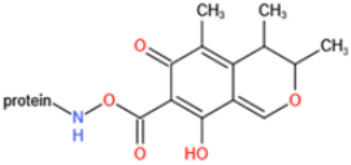 CIT-BSA	Activated ester method	mAb	32,000	0.28 ng/mL	0.01 ng/mL	[164]
PAT	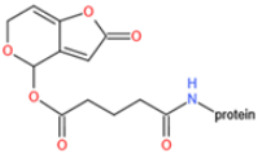 PAT-HG-BSA	Carbodiimide method	pAb	1100	n.a.	n.a.	[165]
	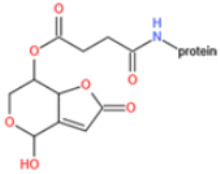 PAT-Ins-HS-BSA ^3^	Carbodiimide method	pAb	n.a.	n.a.	10 ng/mL	[166]
	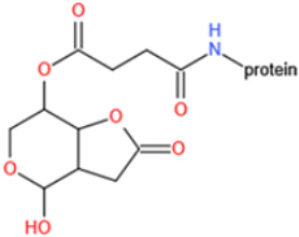 PAT-Sat-HS-BSA ^4^	Carbodiimide method	pAb	n.a.	n.a.	10 ng/mL	[166]

n.a., data not available; ^1^ HG, hemiglutarate; ^2^ HS, hemisuccinate; ^3^ PAT-Ins-HS, 4-[(4-Hydroxy-2-oxo-2,6,7,7a-tetrahydro-4H-furo[3,2-c]pyran-7-yl)oxy]-4-oxobutanoic acid; ^4^ PAT-Sat-HS, 4-[(4-Hydroxy-2-oxohexahydro-4H-furo [3,2-c]pyran-7-yl)oxy]-4-oxobutanoic acid.

**Table 4:** Sequences and dissociation constant (K_D_) of commonly used mycotoxin aptamers.

Target	Sequence (5′-3′)	K_D_	Reference
AFB1	GT TGG GCA CGT GTT GTC TCT CTG TGT CTC GTG CCC TTC GCT AGG CCC ACA	n.a. *	[181]
AFM1	ACT GCT AGA GAT TTT CCA CAT	n.a.	[190]
OTA	GAT CGG GTG TGG GTG GCG TAA AGG GAG CAT CGG ACA	0.2 μM	[180]
FB1	ATA CCA GCT TAT TCA ATT AAT CGC ATT ACC TTA TAC CAG CTT ATT CAA TTA CGT CTG CAC ATA CCA GCT TAT TCA ATT AGA TAG TAA GTG CAA TCT	100 ± 30 nM	[191]
ZEN	TCATCTATCTATGGTACATTACTATCTGTAATGTGATATG	41 ± 5 nM	[192]
DON	GCATCACTACAGTCATTACGCATCGTAGGGGGGATCGTTAAGGAAGTGCCCGGAGGCGGTATCGTGTGAAGTGCTGTCCC	n.a.	[185]
PAT	GGCCCGCCAACCCGCATCATCTACACTGATATTTTACCTT	21.83 ± 5.022 nM	[187]
T-2	GTATATCAAGCATCGCGTGTTTACACATGCGAGAGGTGAA	20.8 ± 3.1 nM	[188]
Ergot alkaloids	ACTCATCTGTGAAGAGAAGCAGCACAGAGGTCAGATGTCCGTCAGCCCCGATCGCCATCCAGGGACTCCCCCCTATGCCTATGCGTGCTACCGTGAA	44 nM ^2^	[189]

* n.a., data not available.

**Table 5:** Commercial products for bioanalytical determination of mycotoxins.

**Mycotoxin**	U.S.: Company/location/website *						
	**Eurofins Abraxis** **Warminster, PA** **USA** **abraxis.eurofins-technologies.com**	**Neogen** **Lansing, MI** **U.S.** **www.neogen.com**	**Beacon Analytical Systems, Inc.** **Saco, ME** **USA** **www.beaconkits.com**	**Envirologix Inc.** **Portland, Main** **USA** **www.envirologix.com**	**Charm Sciences, Inc.** **Lawrence, MA** **USA** **www.charm.com**	**Vicam** **Milford, MA** **USA** **www.vicam.com**	**Romer Labs, Inc** **Newark, DE** **USA** **www.romerlabs.com**
AFB1	n.a.	n.a.	n.a.	n.a.	n.a.	IAC	ELISA, LFD
AFB2	n.a.	n.a.	n.a.	n.a.	n.a.	IAC	n.a.
AFG1	n.a.	n.a.	n.a.	n.a.	n.a.	n.a.	n.a.
AFG2	n.a.	n.a.	n.a.	n.a.	n.a.	n.a.	n.a.
Total AFs	ELISA	ELISA, IAC, LFD	ELISA (plate, tube)	LFD	LFD	IAC, LFD	ELISA, LFD
AFM1	ELISA	ELISA, LFD	ELISA	n.a.	LFD	IAC, LFD	ELISA
OTA	ELISA (OTA, B, C)	ELISA, LFD	n.a.	LFD	n.a.	IAC	ELISA, LFD
ZEN	IAC, LFD	n.a.	ELISA (plate, tube)	LFD	LFD	IAC, LFD	ELISA, LFD
DON	ELISA	ELISA, IAC, LFD	ELISA	LFD	LFD	IAC, LFD	ELISA, LFD
FB1	n.a.	n.a.	n.a.	n.a.	n.a.	n.a.	LFD
Total FMs	n.a.	ELISA, LFD	ELISA	LFD	LFD	IAC, LFD	ELISA, LFD
T-2	n.a.	n.a.	ELISA	n.a.	n.a.	IAC, LFD	ELISA
T-2/HT-2	n.a.	ELISA, LFD	ELISA	LFD	LFD	IAC, LFD	n.a.
PAT	n.a.	n.a.	n.a.	n.a.	n.a.	n.a.	n.a.
CIT	n.a.	n.a.	ELISA	n.a.	n.a.	IAC	n.a.
Ergot alkaloids	n.a.	LFD	n.a.	n.a.	n.a.	n.a.	n.a.
**Mycotoxin**	**Europe: Company/location/website**						
	**Biosense Laboratories AS** **Bergen** **Norway** **www.biosense.com**	**R-Biopharm** **Darmstadt** **Germany** **r-biopharm.com**	**Abcam, Inc.** **Cambridge** **U.K.** **www.abcam.com** **(abcam.cn** **abcam.jp)**	**antibodies-online GmbH** **Aachen#** **Germany** **www.antikoerper-online.de**	**Agrisera AG** **Umea** **Sweden** **www.agrisera.com**	**Biomol** **Hamburg** **Germany** **www.biomol.com**	**Aokin AG** **Berlin** **Germany** **www.aokin.com**
AFB1	n.a.	ELISA	mAb	pAb, mAb, ELISA	ELISA	mAb, ELISA, LFD	mAb
AFB2	n.a.	n.a.	n.a.	n.a.	n.a.	mAb	n.a.
AFG1	n.a.	n.a.	n.a.	n.a.	n.a.	mAb	n.a.
AFG2	n.a.	n.a.	mAb	n.a.	n.a.	mAb	n.a.
Total AFs	ELISA	ELISA, LFD, IAC	n.a.	mAb, ELISA	ELISA	mAb, ELISA, LFD	IAC
AFM1	n.a.	ELISA	n.a.	ELISA	pAb	mAb, ELISA, LFD	mAb, IAC
OTA	n.a.	ELISA, IAC	pAb, mAb	ELISA	pAb	mAb, ELISA, LFD	mAb, IAC
ZEN	ELISA	ELISA, LFD, IAC	mAb	pAb, mAb, ELISA	ELISA	mAb, ELISA, LFD	mAb, IAC
DON	ELISA	ELISA, LFD, IAC	pAb	pAb, mAb, ELISA	pAb	mAb, ELISA, LFD	mAb, IAC
FB1	n.a.	ELISA, LFD, IAC	mAb	ELISA	n.a.	mAb, ELISA, LFD	mAb, IAC
Total FMs	ELISA	n.a.	n.a.	n.a.	n.a.	mAb	n.a.
T-2	ELISA	ELISA, IAC	n.a.	ELISA	n.a.	ELISA, LFD	mAb
T-2/HT-2	n.a.	ELISA, LFD, IAC	n.a.	n.a.	n.a.	n.a.	mAb, IAC
PAT	n.a.	MISPE	n.a.	pAb	pAb	n.a.	n.a.
CIT	n.a.	ELISA, IAC	n.a.	n.a.	n.a.	n.a.	n.a.
Ergot alkaloids	n.a.	n.a.	n.a.	n.a.	n.a.	mAb	mAb
**Mycotoxin**	**China: Company/location/website**						
	**Cusabio** **Technology Co., Ltd.** **Wuhan** **cusabio.cn**	**Lvdu Bio-sciences 6 Technology Co., Ltd.** **Binzhou, Shandong** **lvdu.net**	**Jiangsu Suwei** **Microbiological** **Research Co., Ltd.** **Wuxi** **jssuwei.com**	**Beijing WDWK Biotechnology Co., Ltd.** **Beijing** **wdwkbio.com**	**Nankai Biotech Co. Ltd.** **Hangzhou** **nkbiotech.com**	**Beijing KWINBON** **Biotechnology Co., Ltd.** **Beijing** **kwinbon.com**	**Shandong Meizheng** **Bio-Tech Co., Ltd.** **Rizhao, Shandong** **meizhengbio.com**
AFB1	ELISA	mAb, IAC, LFD, ELISA	ELISA, LFD, IAC	ELISA, LFD	LFD	ELISA, LFD, IAC	ELISA, LFD, IAC
AFB2	n.a.	n.a.	n.a.	n.a.	n.a.	n.a.	n.a.
AFG1	n.a.	n.a.	n.a.	n.a.	n.a.	n.a.	n.a.
AFG2	n.a.	n.a.	n.a.	n.a.	n.a.	n.a.	n.a.
Total AFs	ELISA	IAC, LFD	ELISA, IAC	n.a.	n.a.	ELISA	ELISA, LFD, IAC
AFM1	ELISA	mAb, IAC, LFD, ELISA	ELISA, LFD, IAC	ELISA, LFD	LFD	ELISA, LFD	ELISA, LFD, IAC
OTA	ELISA	mAb, IAC, LFD, ELISA	ELISA, LFD, IAC	ELISA, LFD	LFD	ELISA, IAC	ELISA
ZEN	ELISA	mAb, IAC, LFD, ELISA	ELISA, LFD, IAC	ELISA, LFD	LFD	ELISA, LFD, IAC	ELISA, LFD
DON	ELISA	mAb, IAC, LFD, ELISA	ELISA, LFD, IAC	ELISA, LFD	LFD	ELISA, LFD, IAC	ELISA, LFD, IAC
FB1	ELISA	mAb, IAC, ELISA	n.a.	n.a.	n.a.	n.a.	n.a.
Total FMs	n.a.	LFD, ELISA	ELISA	ELISA, LFD	LFD	ELISA, LFD	ELISA, LFD
T-2	n.a.	mAb, IAC, LFD, ELISA	ELISA	ELISA, LFD	LFD	ELISA	ELISA
T-2/HT-2	n.a.	n.a.	n.a.	n.a.	n.a.	n.a.	n.a.
PAT	n.a.	mAb	n.a.	n.a.	n.a.	n.a.	n.a.
**Mycotoxin**	**China: Company/location/website**						
	**Cusabio** **Technology Co., Ltd.** **Wuhan** **cusabio.cn**	**Lvdu Bio-sciences 6 Technology Co., Ltd.** **Binzhou, Shandong** **lvdu.net**	**Jiangsu Suwei** **Microbiological** **Research Co., Ltd.** **Wuxi** **jssuwei.com**	**Beijing WDWK Biotechnology Co., Ltd.** **Beijing** **wdwkbio.com**	**Nankai Biotech Co. Ltd.** **Hangzhou** **nkbiotech.com**	**Beijing KWINBON** **Biotechnology Co., Ltd.** **Beijing** **kwinbon.com**	**Shandong Meizheng Bio-Tech Co., Ltd.** **Rizhao, Shandong** **meizhengbio.com**
CIT	n.a.	IAC	n.a.	n.a.	n.a.	n.a.	n.a.
Ergot alkaloids	n.a.	n.a.	n.a.	n.a.	n.a.	n.a.	n.a.

n.a., information not available. Used abbreviations: mAb, monoclonal antibody; pAb, polyclonal antibody; ELISA, enzyme-linked immunosorbent assay; LFD, lateral flow device; IAC, immunoaffinity chromatography. * All the websites were accessed on 10 December 2021.

**Table 6:** Providers of multiplexed immunochemical analyses systems (biochips/beads).

Provider	Principle	Internet Address *
Luminex Corporation, Austin, TX, USA	suspension assay	www.luminexcorp.com
Becton Dickinson Biosciences, Franklin Lakes, NJ, USA	suspension assay	www.bdbiosciences.com
Quanterix Corp., Lexington, MA, USA	suspension assay	www.Quanterix.com
Merck Millipore, Burlington, MA, USA	suspension assay	www.merckmillipore.com
Bio-Rad-Laboratories, Hercules, CA, USA	suspension assay	www.bio-rad.com
SAFIA Technologies GmbH, Berlin, Germany	suspension assay	www.safia.tech
Foss GmbH, Hamburg, Germany	suspension assay	www.fossanalytics.com
Unisensor Seraing, Belgium	suspension assay	www.unisensor.be
Randox-Laboratories, Crumlin, UK	planar array (biochip)	www.randoxfood.com
GWK Präzisionstechnik GmbH, München, Germany	planar array (biochip)	www.gwk-munich.com

* All websites were last accessed on 10 December 2021.

**Table 7:** Providers of biosensors that are based on different detection principles.

Provider	Principle ^1^	Internet Address *
GE Healthcare, Chicago, IL, USA	SPR	www.biacore.com/lifesciences
Biolin Scientific, Gothenburg, Sweden	QCM	www.biolinscientific.com/qsense
Micromotive GmbH, Mainz Germany	Microcantilever array	www.micromotive.de
2bind GmbH, Regensburg, Germany	Bio-layer-interferometrie	www.2bind.com
Dynamic Biosensors GmbH, München, Germany ^2^	ESB	www.dynamic-biosensors.com

^1^ SPR, surface plasmon resonance; QCM, quartz crystal microbalance; ESB, electro-switchable biosurfaces. ^2^ Antibodies and microchips are available from Technical University Munich. * All websites were last accessed on 10 December 2021.

## Data Availability

Not applicable.
